# Diurnal oscillations of epigenetic modifications are associated with variation in rhythmic expression of homoeologous genes in *Brassica napus*

**DOI:** 10.1186/s12915-023-01735-7

**Published:** 2023-10-31

**Authors:** Zhifei Xue, Baibai Gao, Guoting Chen, Jie Liu, Weizhi Ouyang, Mohamed Frahat Foda, Qing Zhang, Xiwen Zhang, Wei Zhang, Mingyue Guo, Xingwang Li, Bin Yi

**Affiliations:** 1 National Key Laboratory of Crop Genetic Improvement, Hubei Hongshan Laboratory, Huazhong Agricultural University, Wuhan, 430070 Hubei China; 2 Lushan Botanical Garden Jiangxi Province and Chinese Academy of Sciences, Jiujiang, 332900 Jiangxi China; 3 Department of Biochemistry, Faculty of Agriculture, Benha University, Toukh, 13736 Qalyubiyya Egypt; 4 College of Informatics, Huazhong Agricultural University, Wuhan, 430070 Hubei China; 5 National Engineering Research Center of Rapeseed, Huazhong Agricultural University, Wuhan, 430070 Hubei China

**Keywords:** Ddiurnal oscillation, Rhythmic expression, Epigenetic modification, Allopolyploidy, *Brassica napus*

## Abstract

**Background:**

Epigenetic modifications that exhibit circadian oscillations also promote circadian oscillations of gene expression. *Brassica napus* is a heterozygous polyploid species that has undergone distant hybridization and genome doubling events and has a young and distinct species origin. Studies incorporating circadian rhythm analysis of epigenetic modifications can offer new insights into differences in diurnal oscillation behavior among subgenomes and the regulation of diverse expressions of homologous gene rhythms in biological clocks.

**Results:**

In this study, we created a high-resolution and multioscillatory gene expression dataset, active histone modification (H3K4me3, H3K9ac), and RNAPII recruitment in *Brassica napus*. We also conducted the pioneering characterization of the diurnal rhythm of transcription and epigenetic modifications in an allopolyploid species. We compared the evolution of diurnal rhythms between subgenomes and observed that the Cn subgenome had higher diurnal oscillation activity in both transcription and active histone modifications than the An subgenome. Compared to the A subgenome in *Brassica rapa*, the An subgenome of *Brassica napus* displayed significant changes in diurnal oscillation characteristics of transcription. Homologous gene pairs exhibited a higher proportion of diurnal oscillation in transcription than subgenome-specific genes, attributed to higher chromatin accessibility and abundance of active epigenetic modification types. We found that the diurnal expression of homologous genes displayed diversity, and the redundancy of the circadian system resulted in extensive changes in the diurnal rhythm characteristics of clock genes after distant hybridization and genome duplication events. Epigenetic modifications influenced the differences in the diurnal rhythm of homologous gene expression, and the diurnal oscillation of homologous gene expression was affected by the combination of multiple histone modifications.

**Conclusions:**

Herein, we presented, for the first time, a characterization of the diurnal rhythm characteristics of gene expression and its epigenetic modifications in an allopolyploid species. Our discoveries shed light on the epigenetic factors responsible for the diurnal oscillation activity imbalance between subgenomes and homologous genes’ rhythmic expression differences. The comprehensive time-series dataset we generated for gene expression and epigenetic modifications provides a valuable resource for future investigations into the regulatory mechanisms of protein-coding genes in *Brassica napus*.

**Supplementary Information:**

The online version contains supplementary material available at 10.1186/s12915-023-01735-7.

## Background

Throughout evolution, organisms have developed intrinsic biological clock systems to effectively adjust and acclimate to the cyclic alterations in Earth’s environment [1–4]. The core components of the biological clock rhythmically regulate downstream gene expression through transcription-translation negative feedback loops (TTFL) to maintain circadian rhythms of life activities [5–8]. The core biological clock system regulates the circadian rhythm of histone modifications in the genome by controlling the diurnal oscillations of gene expression responsible for initiating histone modifications [9, 10]. Active histone modifications enhance the binding activity of cis-regulatory transcription factors to the genome, facilitating the recruitment of RNAPII to the transcription start sites. This promotes the transcription activation of rhythm genes throughout the genome, thereby maintaining the diurnal oscillation of genome-wide transcription.

Numerous studies have combined research investigations on the circadian rhythm of animals, plants, and microorganisms with high-throughput technologies to explore various perspectives, including transcriptome, histone modification, DNA methylation, transcription factor recruitment, proteome, and metabolism [4]. In mouse liver circadian rhythm studies, mRNA sequencing (RNA-seq) and chromatin immunoprecipitation sequencing (ChIP-seq) identified genome-wide cis-regulatory elements of the core biological clock components [7]. These studies confirmed that histone modifications’ diurnal oscillations can promote the gene expression’s diurnal oscillation [11]. Furthermore, the analysis of RNAPII recruitment and rhythmic gene expression revealed dynamic changes in gene expression during the circadian cycle, and the related epigenetic modification of circadian remodeling [12]. Diurnal RNA-seq experiments in multiple organs revealed circadian transcription heterogeneity across tissues and gene diurnal oscillations’ generality [13]. In *B. rapa*, gene coexpression network analysis of rhythmic genes found that the circadian fluctuation of gene expression is extensively altered under drought stress, thus identifying drought-responsive genes [14]. RNA-seq of diurnal oscillations in soybean revealed that the biological clock responds to abiotic stress by regulating the phase of gene oscillations [15]. Circadian oscillating RNA-seq in Arabidopsis shows a circadian rhythm in clock-regulated alternative splicing [16]. In Arabidopsis, studies of the dynamics of histone modifications have described that H3K27me3 and H3K4me3 exhibit distinct plasticity and stability in diurnal and seasonal fluctuations, respectively [17]. The circadian changes in protein-coding gene expression in plants depend on the dynamic reversible acetylation and phosphorylation of histone modifications in corresponding genomic regions, and reduced binding of repressive transcription factors contributes to activating-property chromatin modifications [18]. Recently, developed technologies such as Chromatin Interaction Analysis with Paired-End Tag (ChIA-PET) and high-throughput chromosome conformation capture (Hi-C) have brought circadian rhythm research into the perspective of three-dimensional space. The circadian rhythm of the three-dimensional genome structure dynamically configures the regulatory elements of gene expression [19–21]. In rice, the circadian dynamics of RNAPII-mediated chromatin interactions correlate with diurnal oscillations in gene expression [22]. Unlike mammals, plants lack the mammalian-like suprachiasmatic nucleus (SCN) of the hypothalamus in which the core circadian oscillator functions [2]. The unique fixed growth pattern and seasonal growth and development characteristics of plants mean that the mechanism by which plants sense periodic changes in the external environment is more sensitive and complex. Similar to the mammalian peripheral clock, the circadian rhythm characteristics of physiological activities in different plant organs are also tissue-specific and related to different light inputs [23]. Chromatin accessibility likewise differs in different Arabidopsis tissues in response to photoperiods [24]. The expression of biological clock genes exhibits tissue specificity with asymmetric coupling [25]. Dispersed plant biological clocks process environmental signals separately in specific tissues, and there is a clear division of labor between biological clocks in each tissue, which coordinates the overall circadian physiological activities of the plant [26]. However, the characterization of diurnal oscillations in epigenetic modifications is still lacking in plant circadian rhythm studies.

Polyploid species typically possess multiple copies of biological clock genes, and elucidating the homologous expression diversity of rhythmic genes can aid in unraveling the complex biological functions in which biological clock genes are involved. The alignment of known biological clock genes between Arabidopsis thaliana and *B. rapa* has revealed that biological clock genes are preferentially retained during gene loss after multiple rounds of genome doubling events [27]. Studies conducted on three different plant morphological *Brassica* species have demonstrated that numerous copies of biological clock genes are selectively retained after a genome doubling event to diversify their functions, thereby contributing to the species diversity of *Brassica* [28]. Atlantic salmon has undergone 4 rounds of whole-genome duplication since vertebrates, and the diversification and tissue-specific expression of multicopy biological clock genes are associated with functions other than the biological clock, such as gill development processes that adapt to migration [29]. Approximately 7500 years ago, the A genome of *Brassica rapa* (*B. rapa*, 2*n*=2x=20, AA) and the C genome of *Brassica oleracea* (*B. oleracea*, 2*n*=2x=18, CC) hybridized naturally and formed *Brassica napus* (*B. napus*, 2*n*=4x=38, AACC) through genome doubling [30, 31]. Due to the imbalance of asymmetric transcription and epigenetic modification, gene expression activity in the An subgenome was significantly higher than that in the Cn subgenome in *B. napus* [32]. The genetic background of allopolyploidy and the recent origin of *B. napus* make it an excellent model for exploring differences in diurnal oscillations between subgenomes and homologous genes.

To characterize the diurnal oscillation characteristics of the *B. napus* genome, we generated high-quality gene expression and histone modification datasets using RNA-seq and ChIP-seq before the onset of flowering. We discovered that the expression of protein-coding genes, histone modifications (H3K4me3, H3K9ac), and RNAPII recruitment exhibited robust diurnal oscillations. While the gene expression and active histone modifications of the An subgenome were higher than those of the Cn subgenome [32], we observed that the diurnal oscillation of the Cn subgenome was more potent than that of the An subgenome. Compared to *B. rapa*, one of *B. napus*’ ancestors, we found extensive changes in the A genome’s circadian oscillatory signature of genes. Furthermore, we found that the differences in diurnal oscillations of expression of homologous genes were associated with changes in various histone modifications during the circadian cycle. Our study analyzes the circadian oscillatory signature of transcription and histone modifications in allopolyploid species, providing insights into genome duplication and the resulting redundancy of biological clock genes, diurnal oscillation differences between subgenomes, and differences in the diurnal oscillation of expression of homologous genes.

## Results

### Robust diurnal oscillation of protein-coding genes in *B. napus*

**Fig. 1:**
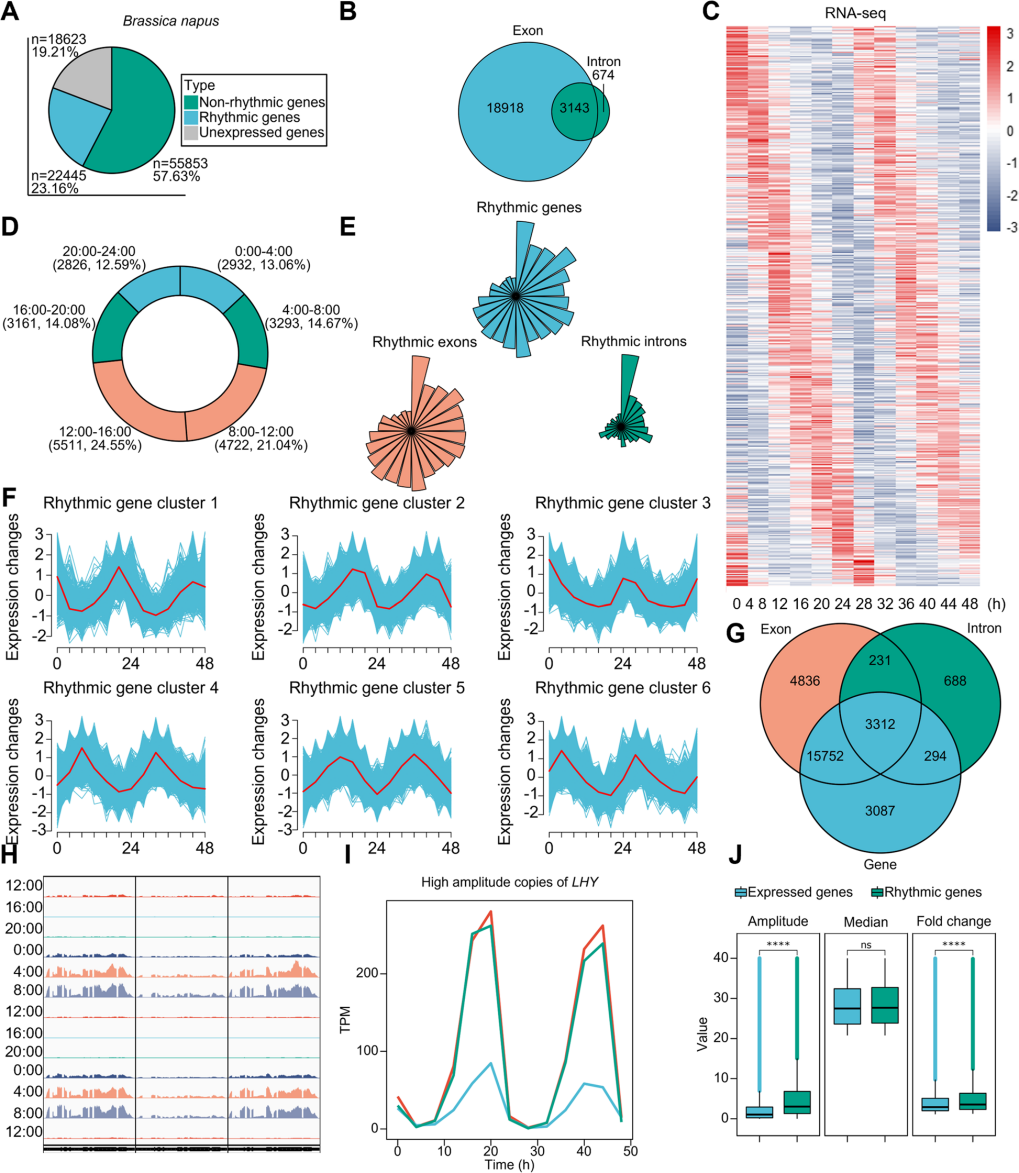
Diurnal oscillation characteristics of protein-coding gene transcription in *B. napus*. **A** Proportions of genes with different expression signatures of protein-coding genes in *B. napus*. **B** The number of genes with overlapping rhythmic exons and introns. **C** The heatmap illustrates the diurnal oscillation of gene expression in rhythmic genes. **D** A frequency plot percentage of rhythmic genes with peak expression phase binned in 4h over 24h is presented. **E** The phase distribution of rhythmic genes, rhythmic exons, and rhythmic introns. **F** Spatial-specific clustering of time-series curves of rhythmic gene transcription. **G** Overlapping genes with oscillating exons and introns overlap with rhythmic genes. **H** Features of gene transcription at the indicated time point of *LHY*. (From left to right, they are *BnaA10G0008600ZS*, *BnaC03G0001300ZS*, and *BnaC05G0010100ZS*) **I** Diurnal fluctuations in high-amplitude homologous gene expression of *LHY*. (The red line represents *BnaA10G0008600ZS*, the blue line represents *BnaC03G0001300ZS*, and the green line represents *BnaC05G0010100ZS*) **J** Comparing amplitude, peak-to-trough ratio, and median of rhythmic gene expression with global gene expression

We collected samples every 4 h over 2 full diurnal cycles under stable meteorological conditions (Additional file 1: Fig. S1) to generate high-quality transcriptomes of *B. napus* by RNA-seq. We aimed to detect the circadian oscillatory signature of protein-coding gene expression. Using one of the latest high-quality reference genome assembly results [33], we mapped 96,921 protein-coding genes in allotetraploid *B. napus* on 10 chromosomes in the An subgenome and 9 chromosomes in the Cn subgenome. To quantify gene expression, we used a deep neural network (DNN)-based machine learning method called BIO_CYCLE [34, 35] and measured transcripts per million (TPM). We detected 22,445 rhythmic genes (23.16%, out of 96,921 protein-coding genes) with *q* values <0.05 and a period of 20 to 28 h (Fig. 1C). Among the protein-coding genes, 18,623 genes (19.21%) were not expressed within 1 week before the flowering transition (Fig. 1A). We also detected 63,016 rhythmic exons (14.00%, of 450,037 exons) and 5491 rhythmic introns (4.96%, of 110,639 introns) at the transcript level (Fig. 1G). We also investigated diurnal oscillations at the transcript level. Throughout the genome, 22,061 genes exhibited diurnal oscillations in their exonic expression, with 97.17% (21,436) classified as rhythmic genes. Additionally, 3817 genes showed diurnal oscillations in their intronic expression, with 86.61% (3,306) classified as rhythmic genes. A total of 3143 genes exhibited diurnal oscillations in both exonic and intronic regions. However, due to variations in the oscillation phases of complex transcripts, 99.52% (3,128) of these are categorized as rhythmic genes (Fig. 1B). Since intronic signals can be interpreted as pre-mRNA expression or nascent transcriptional events [36], and exonic signals as mRNA transcriptional events [7], we believe that the diurnal oscillation of gene expression can be attributed to the diurnal oscillation of exons (Fig. 1E).

We partitioned 24 h into 6 intervals of 4 h and calculated the phase distribution of all rhythmic gene expression peaks. Among them, 45.59% of rhythmic gene expression peaks occurred during the daytime (8:00–16:00), and the ratios of genes expressed during dusk (16:00–20:00) and early morning (4:00–8:00) were similar. Similarly, the ratios of genes expressed during the morning (8:00–12:00) and afternoon (12:00–16:00) were similar, as were the ratios of genes expressed during eve (20:00–24:00) and dawn (0:00–4:00) (Fig. 1D). The median, amplitude, and fold change of rhythmic genes were higher than those of globally expressed genes (Fig. 1J). Gene Ontology (GO) analysis revealed that rhythmic genes had extensive binding and catalytic activities and were involved in biological processes such as metabolism and cellular processes (Additional file 1: Fig. S3C). Kyoto Encyclopedia of Genes and Genomes (KEGG) pathway analysis indicated that most rhythmic genes had kinase activity (Additional file 1: Fig. S3D). Clustering analysis of rhythmic genes based on time-series spatial similarity features fully revealed differences in gene diurnal oscillation features (Fig. 1F).

As an allopolyploid species, *B. napus* typically has multiple copies of genes. *LHY* encodes a critical transcription factor involved in the plant’s biological clock and can bind to the promoter regions of *TOC1* and *CHE* to inhibit their transcription, thereby suppressing the expression of *CCA1*. In *B. napus*, 8 copies of *LHY* are distributed on 6 chromosomes in the An/Cn subgenomes. Among the 5 rhythmic copies of *LHY*, 3 are categorized as high-amplitude rhythmic genes (Fig. 1H, I), while the remaining 2 display lower oscillation amplitudes (Additional file 1: Fig. S3B).

### Epigenetic modifications with activating properties promote diurnal oscillations in gene expression

**Fig. 2:**
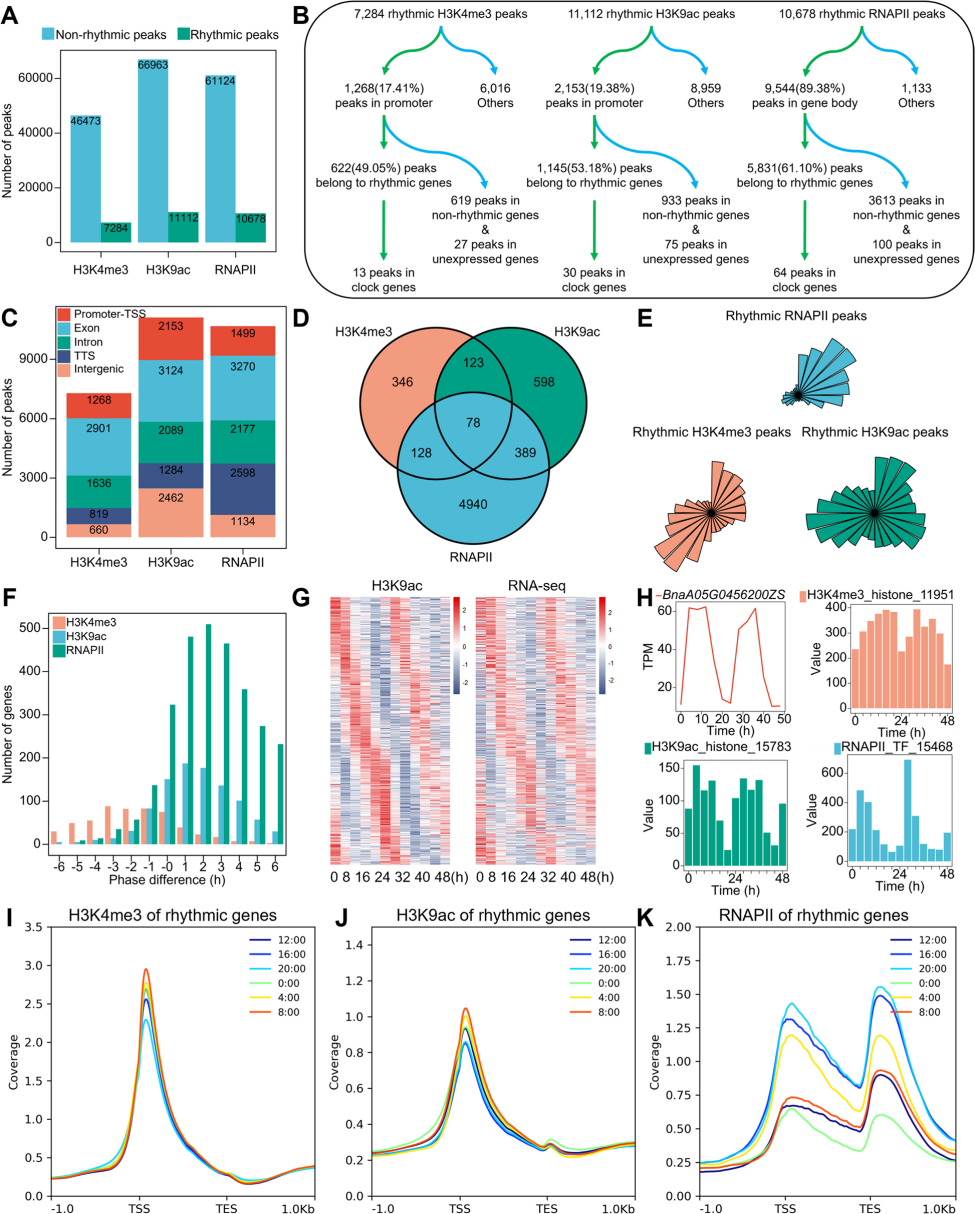
Diurnal oscillation patterns of epigenetic modifications. **A** Ratio of rhythmic peaks in histone modifications H3K4me3, H3K9ac, and RNAPII recruitment. **B** The rhythmic peaks of histone modifications H3K4me3, H3K9ac, and RNAPII recruitment are distributed in different rhythmic feature genes. **C** Annotation of rhythmic peaks for histone modifications H3K4me3, H3K9ac, and RNAPII recruitment. **D** Overlapping genes with rhythmic peaks in histone modifications H3K4me3, H3K9ac, RNAPII recruitment, and rhythmic genes. **E** Phase distribution of rhythmic peaks in histone modifications H3K4me3, H3K9ac, and RNAPII recruitment. **F** Phase differences between rhythmic peaks and overlapping genes of histone modifications H3K4me3, H3K9ac, and RNAPII recruitment. **G** Heatmap of rhythmic peaks modified by histone modification H3K9ac and their gene transcription. **H** Diurnal oscillation of COL9 homologous gene expression and its histone modifications H3K4me3, H3K9ac, and RNAPII recruitment. **I** Diurnal oscillation of histone modification H3K4me3 on rhythmic genes. **J** Diurnal oscillation of histone modification H3K9ac on rhythmic genes. **K** Diurnal oscillations of RNAPII recruitment on rhythmic genes

We produced a collection of circadian rhythmic histone modifications data, encompassing H3K4me3 and H3K9ac, both linked to the activation of gene expression, along with RNAPII recruitment, a participant in gene expression, within *B. napus* under identical conditions to those of the diurnally fluctuating transcriptome dataset. We observed robust diurnal oscillation characteristics for histone modifications using the same diurnal oscillation detection method as the transcriptome dataset (Fig 2I–K). We compared histone modifications’ dynamic abundance at expressed, non-rhythmic, and non-expressed genes (Additional file 1: Fig. S6C-E; S7C-E; S8C-E). Furthermore, we compared the differences in histone modifications between rhythmic and non-rhythmic genes at the same time, as well as between expressed genes and non-expressed loci (Additional file 1: Fig. S6G, H; S7G, H; S8G, H). Although histone modifications are related to gene expressional activation, their phase distributions were significantly different (Fig. 2E). Moreover, the clustering analysis of diurnal oscillation variations revealed distinctions in temporal sequence features and oscillatory characteristics (Additional file 1: Fig. S5A-C).

We identified 53,738 H3K4me3 peaks throughout the genome, of which 13.55% (7284) were diurnal oscillations (Fig. 2A, B). Peak annotation results revealed that 1268 rhythmic H3K4me3 peaks are located within the promoter regions of protein-coding genes (Fig. 2C). There are 622 H3K4me3 peaks located within the promoters of 622 rhythmic genes (Fig. 2B, Additional file 1: Fig. S4A). GO enrichment analysis indicated these genes were mainly associated with metabolic and catalytic activity (Additional file 1: Fig. S4G). For H3K9ac, we detected 78,069 peaks throughout the genome, of which 14.23% (11,112) were diurnal oscillations (Fig 2A, B). Peak annotation results reveal that 2153 rhythmic H3K9ac peaks are within the protein-coding gene promoter regions (Fig. 2B, C). There are 1145 H3K4me3 peaks located within the promoters of 1141 rhythmic genes (Fig. 2B, Additional file 1: Fig. S4B). GO enrichment analysis revealed these genes were broadly binding, catalytically active, and involved in biological processes such as metabolism and cells (Fig. S4I). Activating histone modifications increases RNAPII recruitment to promote gene expression [37]. We identified 71,166 peaks throughout the genome, of which 15.00% (10,678) were diurnal oscillations (Fig 2A, B). Annotation results indicate the presence of 9544 rhythmic RNAPII peaks across the gene bodies of 9265 protein-coding genes (Fig. 2B). Among them, 5600 genes, accounting for 60.44%, were identified as rhythmic genes (Additional file 1: Fig. S4C). GO enrichment analysis revealed that these genes are involved in metabolic and cellular processes, with catalytic and transcription factor binding activities (Additional file 1: Fig. S4K). The peak phase distributions of the histone modifications H3K4me and H3K9ac on diurnal oscillatory peaks were similar, with a higher ratio during the day (8:00–16:00) than at night (20:00–4:00) (Additional file 1: Fig. S4D, E). However, the peak phase of histone modifications by RNAPII recruitment was mainly concentrated at dusk (16:00–20:00) and the eve of the night (20:00–24:00) (Additional file 1: Fig. S4F). The diurnal oscillation of histone modification H3K9ac and RNAPII recruitment preceded that of protein-coding genes (Fig. 2F, G; Additional file 1: Fig. S4J, L; S11D, E). Although associated with the transcriptional activation of genes, the diurnal oscillation of histone modification H3K4me3 peaks occurred later than that of gene expression (Fig. 2F; Additional file 1: Fig. S4H; S11B, C).

The histone modifications H3K4me3 and H3K9ac are associated with the activation of gene expression, while RNAPII directly participates in this process. Among rhythmic genes, 29.41% (6602) exhibited at least 1 type of histone modification with diurnal oscillations (Fig. 2D). As previously reported [11], H3K4me3 and H3K9ac, which facilitate diurnal oscillations in transcription, exhibit robust diurnal oscillations not only in promoter regions, but also in other elements of the genome (Fig. 2C). However, some genes with diurnal oscillation modifications do not express, manifesting as an accumulated expression level of 0 during the day-night cycle (Fig. 2B). Diurnal oscillations of histone modifications are not exclusive to rhythmic genes, but are widespread across the genome. We identified histone modification peaks in a total of 55,038 genes, representing 56.78% of all protein-coding genes. Among these genes, 16,915 have at least 1 type of histone modification peak that oscillates day and night, and the expression of 9351 genes also oscillates. Although H3K4me3 activates promoters, H3K9ac activates promoters and enhancers, and RNAPII is directly involved in transcription, our results demonstrate that the diurnal oscillating histone modifications detected in different regions of protein-coding gene bodies contribute to varying degrees of diurnal oscillations in transcription.

We examined the correlation between histone modification elements and gene expression using Pearson’s correlation coefficient (R). The diurnal oscillation of H3K4me3 modification peaks showed a positive correlation with gene expression diurnal oscillation (*R*=0.54). The correlation coefficient of promoter and exon regions was slightly lower than the global average, while intron regions showed the highest correlation (*R*=0.64), and transcription termination regions were the least correlated (*R*=0.31) (Additional file 2: Supplementary Dataset 1). The diurnal oscillation of H3K9ac modification peaks was also positively correlated with gene expression diurnal oscillation (*R*=0.58), with the correlation coefficient at the promoter region slightly higher than that at the global level (*R*=0.59). Exon regions showed the highest correlation (*R*=0.61), while intron regions had a moderate correlation with transcription termination regions (0.45<*R*<0.46) (Additional file 3: Supplementary Dataset 2). The diurnal oscillation of RNAPII modification peaks was also positively correlated with gene expression diurnal oscillation (*R*=0.51). The correlation coefficients of the promoter region, exon region, intron region, and transcription termination region were all close (0.43<*R*<0.52) (Additional file 4: Supplementary Dataset 3). The diurnal oscillations of epigenetic modifications associated with gene expression activation exhibit varying degrees of correlation with the diurnal oscillations of protein-coding gene expression. Moreover, these correlations display distinctions among different elements. Furthermore, differences in chromatin accessibility were observed between rhythmic and non-rhythmic genes, as well as expressed and non-expressed genes (Additional file 1: Fig. S9A, C). Motif analysis of rhythmic, non-rhythmic, and non-expressed genes revealed differences between discrete gene classes (Additional file 1: Fig. S9B, D, and F).

Using H3K4me3 as a defining mark, we performed a preliminary study to explore the diurnal oscillations of epigenetic modifications on core plant clock genes in *B. napus*, including *COL2*, *COL9*, *GI*, *LNK1*, *PIF4*, *PRR3*, *PRR7*, *RVE4*, *SPA1*, and *TIC*. H3K9ac is also involved in modifying the promoter regions of core clock genes, including *CBF2*, *CCR1*, *CDF2*, *COL9*, *COR27*, *COR28*, *GI*, *HYH*, *LNK1*, *LNK2*, *PHYB*, *PIF4*, *PRR3*, *PRR5*, *PRR7*, *PRR9*, *RVE1*, *RVE2*, *RVE3*, *RVE4*, and *TIC* (Additional file 5: Supplementary Dataset 4). *COL9*, which belongs to the *CO* gene family and regulates the expression of *FT* and *SOC1*, has 5 homologous copies. We observed that the promoter region of *BnaA05G0456200ZS* exhibits diurnal oscillations in both H3K4me3 peaks (*R*=0.42) and H3K9ac peaks (*R*=0.80) (Fig. 2H).

### Diurnal oscillation of the Cn subgenome exhibits a stronger amplitude than that of the An subgenome

**Fig. 3:**
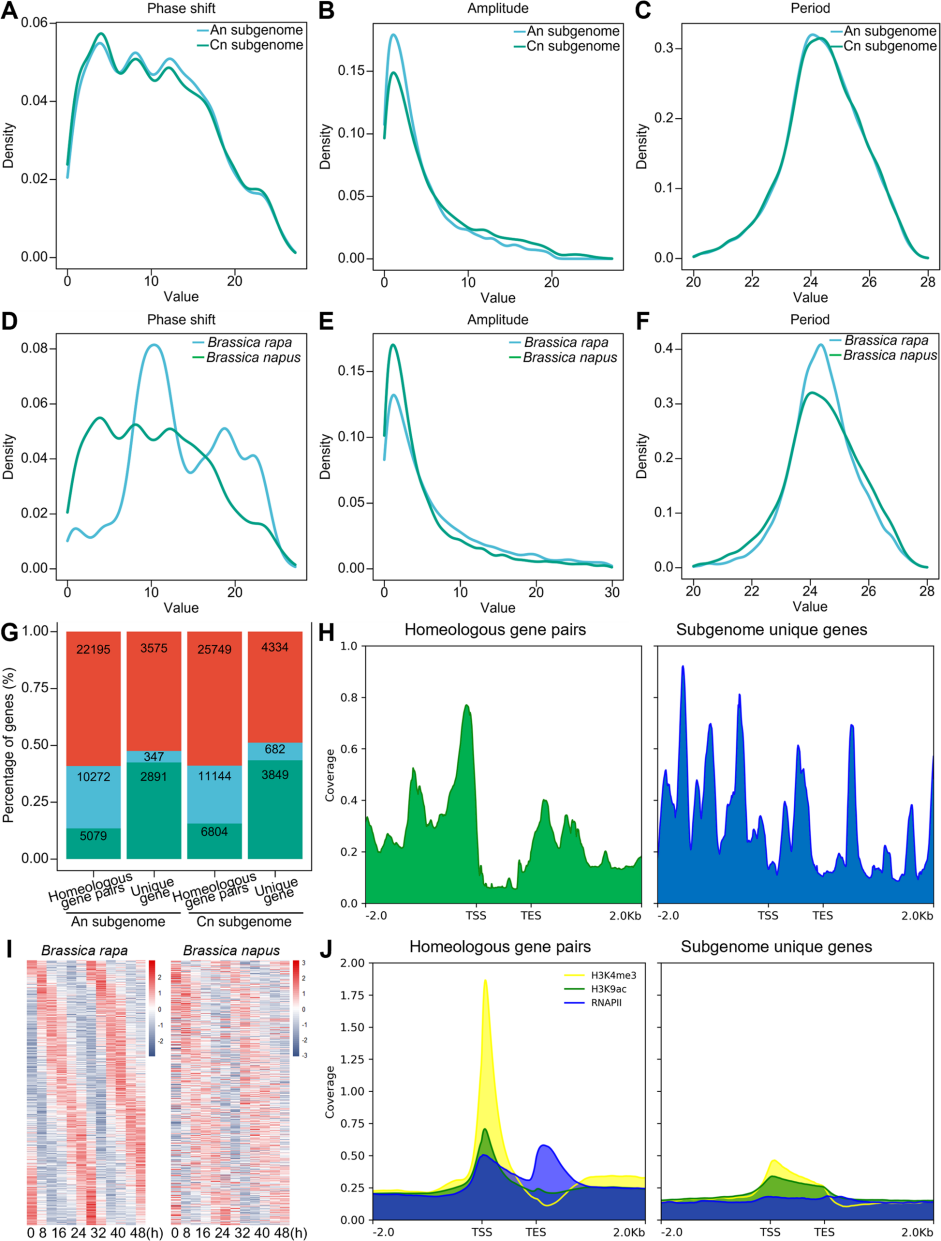
Diurnal oscillation disparities between subgenomes and the evolution of their characteristics. **A–C** Density distributions of phase shifts, amplitudes, and periods of rhythmic genes in the An/Cn subgenome of *B. napus*. **D–F** Density distributions of phase shifts, amplitudes, and periods of rhythmic genes in the *B. rapa* A genome and the *B. napus* An subgenome are presented. **G** The proportion of genes with different expression characteristics between homologous gene pairs and subgenome-specific genes. (Red represents non-rhythmic genes, blue represents rhythmic genes, and green represents unexpressed genes) **H** Chromatin accessibility disparities between homologous genes and subgenome-specific genes. **I** The expression heatmap of rhythmic genes in the *B. rapa* A genome and homologous genes in the *B. napus* An subgenome. **J** Differences in histone modifications between homologous genes and subgenome-specific genes

We compared the oscillatory properties of rhythmic genes between the An/Cn subgenomes of *B. napus*. Our findings suggest that the rhythmic genes on the An/Cn subgenomes had similar distribution densities in phase shift, amplitude, and period (Fig. 3A–C). While there was a significant difference in the distribution of expressed genes between the An/Cn subgenomes, there was no difference in the distribution of rhythmic genes (Additional file 1: Fig. S9G). Integrating the epigenetic modification dataset, we observed differential diurnal oscillation strengths in active epigenetic modifications between the An/Cn subgenomes. Moreover, the diurnal oscillation peaks of epigenetic modifications in the Cn subgenome were found to be higher than those in the An subgenome (Additional file 1: Fig. S6A, B, and F; S7A, B, and F; S8A, B, and F). In contrast to the Cn subgenome, the An subgenomes exhibit higher chromatin accessibility at the transcription start sites and upstream regions of rhythmic genes. The presence of large and small peaks suggests the potential existence of multiple regulatory elements, such as enhancers or transcription factor binding sites (Additional file 1: Fig. S9E).

We compared the differences in rhythmic genes between two *Brassica* species, *B. napus* and *B. rapa*, explicitly focusing on the A genome. We used the published diurnal oscillation dataset from the natural environment and applied the same diurnal oscillation detection method as in *B. napus* [14, 38]. The An subgenome of *B. napus* contains 44,359 genes, of which 36,389 are expressed, and 10,619 are rhythmic. The A genome of *B. rapa* has 45,409 genes, of which 36,344 are expressed, and 15,314 are rhythmic. We also compared the changes in the An subgenome’s rhythmic gene day-night oscillation characteristics during the evolution from *B. rapa* to *B. napus*. The phase shift of rhythmic genes showed extensive variation (Fig. 3D), indicating that after encountering the A/C genomes, functional gene redundancy and chromosomal variations affected the temporal expression patterns of most genes. While the density distribution curves of the amplitudes and periods of rhythmic genes in *B. rapa* and *B. napus* showed certain similarities, the distantly hybridized and genome-duplicated events led to a noticeable trend of higher numbers of low-amplitude genes and reduced numbers of high-amplitude genes (Fig. 3E). The oscillation periods of rhythmic genes also exhibited a trend of differentiation, either shorter or longer than 24 h (Fig. 3F). We aligned the protein sequences of rhythmic genes in *B. rapa* with their homologous genes in the An subgenome of *B. napus*. The diurnal oscillatory heatmap of gene expression showed phase advancement and widespread diurnal oscillatory pattern changes (Fig. 3I). The proportion of genes expressed in An subgenome of *B. napus* during the day-night cycle was significantly higher than that of *B. rapa*. In comparison, the proportion of rhythmic genes was substantially lower than that of the A genome of *B. rapa* (Additional file 1: Fig. S9H).

The encounter of the A genome of *B. rapa* and the C genome of *B. oleracea*, prior to subsequent polyploidization in *B. napus*, resulted in the redundancy of functional genes. The gene dosage hypothesis suggests that the functions of homologous genes in *B. napus* would produce a trend of diversification [39]. To investigate this, we constructed a list of homologous genes across 4 species: *Arabidopsis thaliana*, *B. rapa*, *B. oleracea*, and two subgenomes of *B. napus*, based on the protein sequences of Arabidopsis protein-coding genes. The protein-coding genes of *B. napus* were divided into subgenome-homologous gene pairs and subgenome-specific genes. We compared the diurnal oscillations of these two groups and found that the proportion of subgenome-homologous gene pairs in the middle rhythmic gene was as high as 40%. In contrast, the proportion of rhythmic genes in the subgenome-specific genes was only 3%, with the latter having a large proportion of genes that were not expressed (Fig. 3G). Differences in chromatin accessibility of homologous genes versus subgenome-specific genes were observed in the ATAC-seq data from the seedling stage of *B. napus* (Fig. 3H). Furthermore, combined with ChIP-seq, we found that the promoter activation-associated histone modifications H3K4me3 and H3K9ac, as well as the RNAPII recruitment involved in transcription, had higher modification peaks on homologous gene pairs (Fig. 3J; Additional file 1: Fig. S11A).

### Alteration of diurnal oscillatory characteristics of biological clock homologous genes during allopolyploidization

We searched for 77 genes directly involved in the core biological clock regulatory network of *Arabidopsis thaliana*. Homologous alignment of protein sequences was used to blast for homologous copies of biological clock genes in *B. rapa*, *B. oleracea*, and *B. napus*. There are 146 homologous circadian genes in the *B. rapa* genome, 170 biological clock homologous genes in the *B. oleracea* genome, and 312 biological clock homologous genes in the *B. napus* genome. The allopolyploid species *B. napus* inherited all homologous circadian genes from *B. rapa* and *B. napus* (Additional file 5: Supplementary Dataset 4). However, after distant hybridization and polyploidization, the copy number of core biological clock homologous genes in *B. napus* varied. For instance, the biological clock genes *ELF4* and *CDF6* have several homologous copies in *B. rapa* and *B. oleracea* but are entirely lost in *B. napus*. Compared to *B. rapa* and *B. oleracea*, the copy numbers of some biological clock homologous genes in *B. napus* have changed due to duplication and deletion. *LHY* has 3 consecutive homologous copies on chromosome C7 and one copy on chromosome C8 in *B. rapa*, whereas in *B. napus*, 1 copy was missing on chromosome C7, and one copy was added adjacent to the homologous gene on chromosome C8. The variation in chromatin structure in *B. napus* has also led to the translocation of biological clock homologous genes between chromosomes. *LWD1* has a copy on chromosome C5 of *B. oleracea*, but in *B. napus*, it produces two copies located on chromosomes C6 and C7. Interestingly, the biological clock genes *ZTL*, *FKF1*, and *LUX-like* were deleted in all three *Brassica* species. In summary, the majority of biological clock homologous genes in *B. napus* are inherited conservatively, both in terms of chromosomal location and quantity, from their counterparts in *B. rapa* and *B. napus*.

In our circadian transcription dataset, 173 out of 312 biological clock homologous genes in *B. napus* exhibit diurnal oscillation properties. Among these genes, 17 homologous copies of biological clock genes lose their circadian rhythm entirely in *B. napus*, while another 60 biological clock genes maintain their transcriptional circadian rhythms, with at least 1 copy of these genes exhibiting diurnal oscillations. To further investigate the diurnal oscillation characteristics of biological clock genes in *B. rapa* and *B. napus*, we compared the expression patterns of homologous copies of these genes in the 2 species using published circadian rhythm transcriptome datasets. Our findings reveal that homologous copies of biological clock genes, demonstrating diurnal oscillations, frequently exhibit distinct amplitudes while maintaining similar phases (Additional file 1: Fig. S10).

### Differential epigenetic modifications contribute to variations in the rhythmic expression of homologous genes

Among the copies of homologous genes in the biological clock of *B. napus*, there were differences in the characteristics of diurnal oscillations, such as amplitude, phase, and expression level. Additionally, we observed differences in the histone modification patterns among the copies of clock genes.

**Fig. 4:**
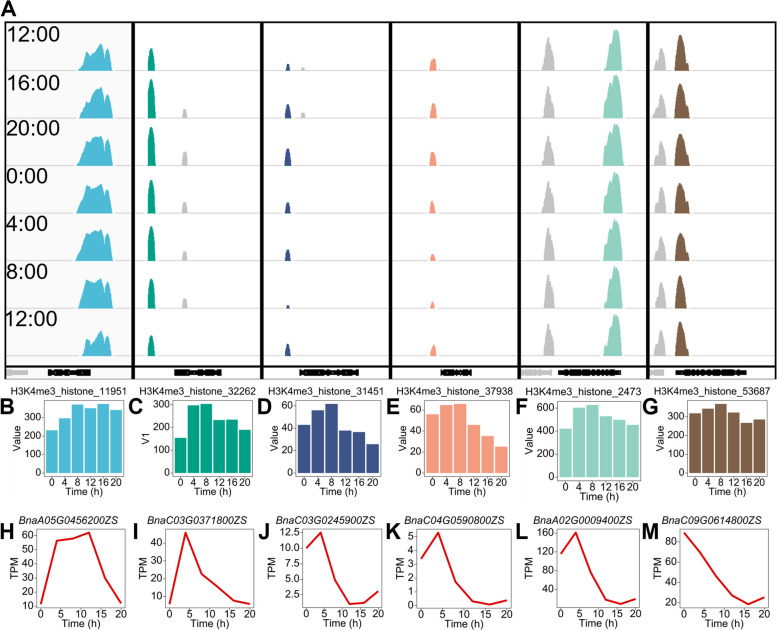
Variation in rhythmic expression of biological clock homologous genes is associated with differences in diurnal oscillations of histone modification H3K4me3. **A** Features of diurnal oscillation of histone modification H3K4me3 on *COL9* (*BnaA05G0456200ZS*, *BnaC03G0371800ZS*), *PIF4* (*BnaC03G0245900ZS*, *BnaC04G0590800ZS*), and *PRR7* (*BnaA02G0009400ZS*, *BnaC09G0614800ZS*). **B–G** Diurnal oscillation of histone modification H3K4me3 on *COL9* (*BnaA05G0456200ZS*, *BnaC03G0371800ZS*), *PIF4* (*BnaC03G0245900ZS*, *BnaC04G0590800ZS*), and *PRR7* (*BnaA02G0009400ZS*, *BnaC09G0614800ZS*). **H–M** Diurnal fluctuations in homologous gene expression of *COL9* (*BnaA05G0456200ZS*, *BnaC03G0371800ZS*), PIF4 (*BnaC03G0245900ZS*, *BnaC04G0590800ZS*), and *PRR7* (*BnaA02G0009400ZS*, *BnaC09G0614800ZS*)

The expression of all 5 homologous genes of *COL9* in *B. napus* exhibits diurnal oscillations. Diurnal oscillation modification signals for *BnaA05G0456200ZS* and *BnaC03G0371800ZS* are located in the promoter region, and the transcriptional diurnal oscillations of these 2 homologous genes differ (Fig. 4H, I). This difference is associated with H3K4me3 (Fig. 4B, C). *PIF4* is a negative regulator of the plant red light response and participates in plant shade avoidance. There are 4 homologous copies of *PIF4* in *B. napus*, and the expression of 3 copies oscillates diurnally. We detected diurnal oscillations of H3K4me3 modification in the promoter regions of *BnaC03G0245900ZS* and *BnaC04G0590800ZS* (Fig. 4D, E), which maintained a similar phase to the diurnal oscillations of gene expression but had differences in amplitude (Fig. 4J, K). *PRR7* is a functionally redundant component of the temperature-sensitive circadian system. *CCA1* and *LHY* positively regulate its expression, and it acts as a transcriptional repressor of both genes, regulating hypocotyl elongation through the photoperiodic pathway. There are 5 homologous copies of *PRR7* in *B. napus*, and the expression of 4 genes oscillates around the clock. We detected diurnal oscillations of H3K4me3 modification in the promoter regions of *BnaA02G0009400ZS* and *BnaC09G0614800ZS* (Fig. 4F, G). The histone modification signal of *BnaA02G0009400ZS* was approximately twice that of *BnaC09G0614800ZS*.

*HYH* is involved in the red light response of plant photosynthesis. All 6 homologous genes of *HYH* in *B. napus* exhibit diurnal oscillation, and histone modification H3K9ac signals that oscillate day and night were detected. The modification signals of *BnaA01G0333200ZS*, *BnaA05G0383200ZS*, and *BnaC01G0412000ZS* are located in the promoter region (Additional file 1: Fig. S12A). The diurnal oscillations of gene transcription are highly correlated with the diurnal oscillations of histone modification signals (Additional file 1: Fig. S12B-D, I-K). The transcription factor encoded by *RVE1* regulates hypocotyl elongation by controlling the auxin level during a specific period. The expression of all 6 homologous genes of this gene in *B. napus* also exhibits diurnal oscillation. The promoter regions of *BnaA10G0195900ZS* and *BnaC02G0079400ZS* exhibit diurnal oscillations of histone modification H3K9ac signals, and the diurnal oscillations of gene expression are highly correlated with the diurnal oscillations of histone modification signals (Additional file 1: Fig. S12E, F, L, and M). *RVE3* is involved in the blue light response, and 3 homologous genes of *RVE3* are found in *B. napus*, namely *BnaA10G0004600ZS*, *BnaA10G0004700ZS*, and *BnaC05G0006800ZS*, all of which exhibit diurnal oscillation and function as circadian rhythm genes. However, it should be noted that *BnaA10G0004600ZS* shows relatively low cumulative expression levels. There was a diurnal oscillation of histone modification H3K9ac signals in the promoter region of the gene, and the diurnal fluctuation of gene expression was highly correlated with the diurnal oscillation of histone modification signals (Additional file 1: Fig. S12G, H, N, and O).

*CHE* has 6 homologous genes in *B. napus*, 5 exhibiting diurnal oscillation in transcription. We detected oscillatory recruitment signals of RNAPII at the transcription start sites of 3 of these genes (Additional file 1: Fig. S13B, C, H, and I). *LHY* has 8 homologous genes in *B. napus*, 5 exhibiting diurnal oscillation in transcription. We also detected oscillating RNAPII recruitment signals at the transcription start sites of 2 of these genes (Additional file 1: Fig. S13D, E, J, and K). Similarly, all 6 homologous genes of *RVE1* in *B. napus* exhibit diurnal oscillation in transcription, and we detected oscillatory RNAPII recruitment signals at the transcription start sites of 3 of these genes (Additional file 1: Fig. S13F, G, L, and M). Although RNAPII is involved in gene expression, we did not detect RNAPII recruitment signals at any diurnally oscillating gene loci. However, for a small portion of diurnally oscillating genes, there was a high correlation between the diurnal oscillations and RNAPII recruitment signals.

### Diverse epigenetic modifications define diurnal oscillations of gene expression

**Fig. 5:**
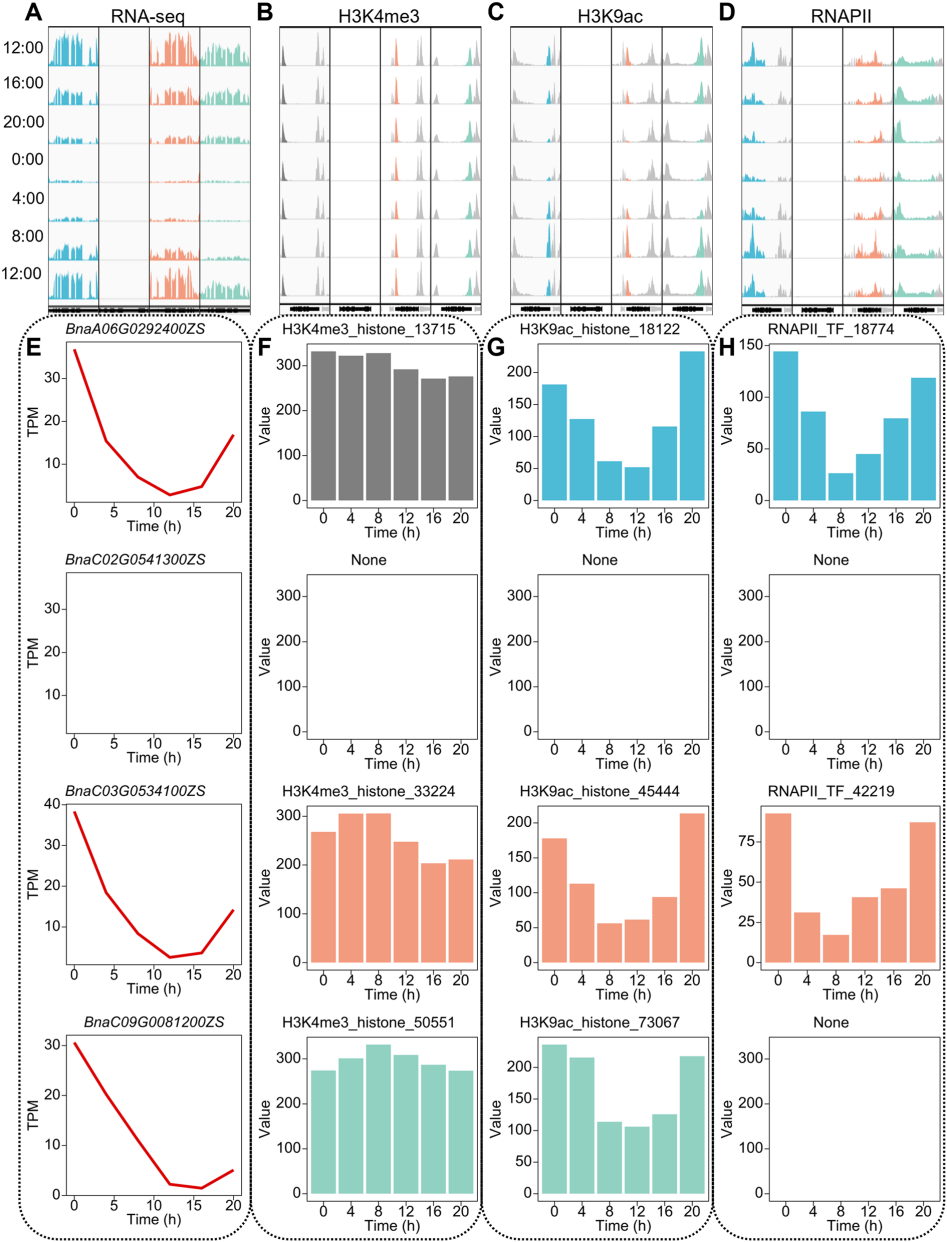
Combinations of multiple epigenetic modification define the diurnal oscillations of gene expression. **A** Features of diurnal gene expression oscillation in 4 homologous genes of *LNK1*. **B** Features of diurnal histone modification H3K4me3 oscillation in 4 homologous genes of *LNK1*. **C** Features of diurnal histone modification H3K9ac oscillation in 4 homologous genes of *LNK1*. **D** Features of diurnal RNAPII recruitment oscillation in 4 homologous genes of *LNK1*. **E** Diurnal oscillation of expression in 4 homologous genes of *LNK1*. **F** Diurnal oscillation of histone modification H3K4me3 in 4 homologous genes of *LNK1*. **G** Diurnal oscillation of histone modification H3K9ac in 4 homologous genes of *LNK1*. **H** Diurnal oscillation of RNAPII recruitment in 4 homologous genes of *LNK1*

The genome harbors a wide array of histone modifications, with various combinations often observed at a specific locus. The diurnal oscillation of rhythmic gene expression is occasionally highly correlated with more than one oscillation of histone modifications. We integrated a table of histone modification data for all protein-coding genes (Additional file 6: Supplementary Dataset 5). When multiple diurnal oscillation histone modifications were identified on rhythmic genes, different types of histone modifications typically exhibited similar diurnal oscillation phases. In conclusion, numerous histone modifications may simultaneously affect the transcription of rhythmic genes. LNK1 plays a role in integrating plant light signaling and the biological clock, which is regulated by the TOC1 complex and acts as a transcriptional coactivator. In *B. napus*, there are 4 homologous copies of *LNK1*, of which *BnaC02G0541300ZS* is a non-expressed gene (Fig. 5E), and no peaks of histone modifications were detected for this gene (Fig. 5F–H). *BnaA06G0292400ZS*, *BnaC03G0534100ZS*, and *BnaC09G0081200ZS* are rhythmic genes (Fig. 5E). Among them, we observed diurnal oscillations of histone modification H3K9ac and RNAPII recruitment signals on *BnaA06G0292400ZS* (Fig. 5G, H), while histone modification H3K4me3 signals displayed diurnal oscillations (Fig. 5F). The expression of this gene exhibits a higher correlation with the recruitment signals of RNAPII during day-night oscillations. The histone modifications H3K4me3 and H3K9ac on *BnaC03G0534100ZS* both undergo day-night oscillations. However, the diurnal gene expression variation correlates more with the day-night oscillations of histone modification H3K9ac (Fig. 5F–H). Despite the day-night oscillations of histone H3K4me3 and H3K9ac modifications on BnaC09G0081200ZS, the oscillations of H3K4me3 are out of phase with gene expression (Fig. 5F). At the same time, while there is a higher correlation between the day-night oscillations of H3K9ac and transcription (Fig. 5G), while RNAPII signals were not detected in *BnaC09G0081200ZS* (Fig. 5H).

We observed a complex combination of histone modifications on *RVE1*. Diurnal oscillations of both H3K4me3 and H3K9ac modifications were detected on all 6 homologous genes of *RVE1* in *B. napus* (Additional file 1: Fig. S14). The diurnal oscillations in the expression of all 6 homologous genes were highly correlated with the diurnal oscillations of H3K9ac modification. We observed that the peak of histone modification H3K4me3 occurs later than the peak of gene expression. In contrast, the peak of histone modification H3K9ac occurs earlier than the peak of gene expression, which is consistent with the results from the genomics analysis section (Fig. 2G). Only 4 copies showed circadian signals of RNAPII recruitment, which were also highly correlated with the diurnal oscillation of gene expression.

## Discussion

Our study presents a high-resolution, multiperiod time-series dataset from *B. napus* that characterizes diurnal oscillations of multiple histone modifications associated with global transcription and gene expression activation in allopolyploid plants. Diurnal oscillations of gene expression play a crucial role in enabling plants to regulate their physiological activities and successfully adapt to the cyclical changes in the Earth’s environmental circadian cycle. Diurnal oscillations of histone modifications contribute to the diurnal oscillation of genes at the transcriptional level, and the superposition of multiple histone modifications jointly enhances the robustness of diurnal oscillations of gene expression. This mechanism enables plants to achieve superior stability in regulating their life activities and successfully adapt to the changes in the external circadian environment [1, 40, 41]. During the process of interspecific hybridization and allopolyploidy, the global circadian rhythm of the genome undergoes alterations. This transformation, coupled with favorable mutations and functional redundancy of core clock genes, bestows new physiological and metabolic advantages upon the resulting hybrids [29, 42].

Although histone modification is a critical factor affecting gene expression activity, gene expression regulation is influenced by multiple factors. Therefore, in our study, we performed chromatin immunoprecipitation experiments only on the histone modifications H3K4me3 and H3K9ac, which have been previously reported to be most strongly associated with diurnal oscillations [7, 11, 12, 22]. We could not detect diurnal oscillatory signals of our target histone modifications at some rhythmic loci, which may suggest that other histone modifications also affect rhythmic gene expression [11, 18]. While both H3K4me3 and H3K9ac are thought to contribute to gene expressional activation by modifying the promoter regions of target genes, we observed circadian oscillating modification signals in regions other than promoters on rhythmic genes. These signals showed a high correlation with diurnal oscillations in gene expression.

Additionally, we found significant differences in chromatin accessibility between different types of gene sets by ATAC-seq experiments, suggesting that the spatial structure of the genome also impacts the circadian rhythm of gene expression. The diurnal oscillation characteristics of gene expression and histone modifications may vary in different plant growth and development stages, and their dynamic relationship warrants further investigation. Notably, the diurnal oscillation of histone modifications of activation properties does not always correspond with the circadian oscillation of gene expression. This phenomenon may be related to the regulation of complex biological processes involved in the biological clock and the spatial structure of the genome.

As a young allopolyploid species with a well-established history of origin, *B. napus* provides an opportunity to explore subgenome-level differences in circadian rhythms. We found that the diurnal oscillation characteristics of gene expression in An subgenome of *B. napus* differ from those of one of its ancestors, A genome of *B. rapa*. The hybridization and polyploidization processes can alter the diurnal oscillation characteristics of the subgenome. While the gene expression level of the An subgenome was superior to that of the Cn subgenome due to asymmetric transcription and the epigenome, the transcriptional and active histone modification signals of rhythmic genes in the Cn subgenome of *B. napus* were higher than those in An subgenome. Future studies on circadian rhythms in *B. oleracea* may provide an in-depth analysis of the C genome’s diurnal oscillation characteristics and evolution.

Generating *B. napus* species involves distant hybridization and subsequent polyploidy of *B. rapa* and *B. napus*, resulting in a complex genetic background and abundant gene redundancy events [31]. In our study, we detected differential histone modification types and distinct peaks at loci of homologous genes with specific diurnal oscillation characteristics such as phase and signal intensity. Differences in diurnal oscillations at the transcriptional level between homologous genes were associated with differences in histone modifications. We propose that the diversity of histone modifications underlies the diversity of homologous expression of rhythmic genes. However, we also found that multiple histone modifications exist at the locus, and different histone modifications sometimes exhibit distinctive diurnal oscillation characteristics. Correlation analysis based on statistics alone cannot determine the causal relationship between diurnal oscillations of gene expression and various histone modifications. Typically, histone modifications are considered to affect gene expression. Our study also confirms that the diurnal oscillation of histone modifications promotes the diurnal oscillation of gene expression. However, the phase of the oscillation of histone modification H3K4me3, which is also associated with gene activation, lags behind the diurnal oscillation of gene expression. The relationship between histone modifications and gene expression in circadian studies remains to be determined.

## Conclusions

Overall, we have generated a dataset of circadian oscillating epigenetic modifications in an allopolyploid species, providing a comprehensive analysis of the circadian oscillatory signatures of gene expression and histone modifications in *B. napus*. We explored intersubgenome differences in the circadian oscillatory behavior of homologous gene expression and found them associated with differences in epigenetic modifications. Our work has bridged a crucial gap in studying circadian rhythms in polyploid species and plants and understanding the diverse impacts of various circadian conditions on plant growth and development. We hope our dataset will pique the interest of researchers investigating functional genes in *B. napus*, and serve as a valuable reference point.

## Methods

### Plant materials and growth conditions

The *B. napus* materials used in this study were mainly sampled before and after flower conversion in the natural environment, and from hydroponic seedlings in the light culture room. Diurnal transcription and chromatin modification experiments were conducted on *B. napus* plants grown under natural conditions. The plants were grown in a field test located at Huazhong Agricultural University School in Hongshan District, Wuhan City, Hubei Province. The seeds were sown on October 1, 2018, and the sampling time before flower conversion was November 22 to 24 (including 2 day and night cycles). Samples were collected from the third intact young leaf from the top of the main stem, which was similar in size and healthy shape. The materials were confirmed to be free of diseases, insect pests, and yellow wilt. For RNA-seq and ChIP-seq experiments, leaves were split in half after removing the central veins. The samples used for RNA-seq experiments were immediately frozen in liquid nitrogen and stored at −80°C. The samples used for ChIP-seq experiments were washed with 1× PBS solution, double-crosslinked, and transferred to a −80 °C freezer. The field test site at Huazhong Agricultural University is located on the Jianghan Plain, which has a humid subtropical monsoon climate with abundant rainfall, sufficient sunshine, and 4 distinct seasons.

Hydroponic seedlings of *B. napus* were grown in a light culture room and were used for the chromatin opening experiment. The seedlings were grown in a hydroponic system in the State Key Laboratory of Crop Genetic Improvement. Samples were collected from the seedlings on the 21st day after sowing, and they were confirmed to be free of diseases, insect pests, and yellow wilt, with consistent morphology and growth momentum. Before performing the ATAC-seq experiment, the samples were washed with 1× PBS solution. The light culture room in the State Key Laboratory of Crop Genetic Improvement was controlled by central air-conditioning to maintain a constant temperature of 24 °C, with a total of 16/8 h of light and dark conditions. The nutrient solution used for hydroponic culture contains 510 mg KNO_3_, 490 mg MgSO_4_·H_2_O, 140 mg KH_2_PO_4_, 1180 mg Ca(NO_3_)_2_.4H_2_O, 13.995 mg FeSO_4_·7H_2_O, 18.61 mg EDTA-2Na, 0.905 mg MgCl_2_·4H_2_O, 0.11 mg ZnSO_4_·7H_2_O, 0.04 mg CuSO_4_·5H_2_O, 1.43 mg H_3_BO_3_, and 0.045 mg Na_2_MoO_4_·4H_2_O.

### RNA-seq and data analysis

Total RNA was extracted from the leaves using the RNeasy Plant Mini Kit from Qiagen. Two micrograms of RNA was used to construct a library using Illumina’s TruSeq Stranded mRNA kit. The resulting library was subjected to paired-end sequencing using Illumina HiSeq X Ten, with a read length of 150 nucleotides per end (PE150). We employed the FastQC software tool to assess the data characteristics of the library. Linkers and low-quality sequences were removed using the Trimmomatic software tool [43]. The library fragments were aligned to the reference genome using the 2-pass mode of Spliced Transcripts Alignment [44]. Uniquely aligned library fragments were retained by calling the view module of Sambamba software using a script [45]. Duplicate sequences were removed from the library using Picard’s MarkDuplicates module. The expression of genes, exons, and introns was calculated using TPMCalculator [46].

### ChIP-seq and data analysis

We cut the clean leaves as much as possible using scissors and immersed them in a 1% formaldehyde solution in 1× PBS. Then, we placed them in a vacuum instrument for cross-linking for 25 min, with vacuum pumping every 5 min in between. We added a 0.2 M glycine solution to stop cross-linking and vacuumed for 5 min. Next, we washed the samples with sterile water 3 times and performed double cross-linking by adding EGS solution (0.02g EGS and 200µl DMSO) and vacuuming again. After finishing, we washed the samples with sterile water 3 times and used filter paper to absorb as much water as possible from the leaf surface. The single- or double-crosslinked materials were used in subsequent experiments or quick-frozen with liquid nitrogen and stored in an ultralow temperature refrigerator at −80 °C for later use. We weighed approximately 1 g of the crosslinked material, ground it thoroughly with liquid nitrogen, and added Buffer S. The mixture was then incubated with rotation at 4 °C for 30 min. Buffer S was composed of 0.05 M HEPES Buffer (pH 7.5), 0.15 M NaCl, 1 mM EDTA, 1% Triton X-100, 0.1% Sodium Deoxycholate, 1% SDS, and 1× PI. We added Buffer F and incubated the mixture for 15 min at 4 °C with rotation. Buffer F was also composed of 0.05 M HEPES Buffer (pH 7.5), 0.15 M NaCl, 1 mM EDTA, 1% Triton X-100, 0.1% Sodium Deoxycholate, and 1× PI. We sonicated the lysate twice to process and interrupt chromatin, with the ultrasonic treatment set for 36 cycles. After each treatment, we centrifuged the supernatant at 12,000 r/min at 4 °C. Then, we mixed an appropriate amount of Protein G magnetic beads with the antibody and incubated them with rotation for 6 to 8 h at 4 °C. Next, we mixed the chromatin fragments obtained after the ultrasonic treatment with Protein G magnetic beads and incubated them for 1 to 3 h to complete the preclearing. After placing the samples on a magnetic stand, we took the supernatant, mixed it with the antibody-incubated Protein G, and incubated it at 4 °C for 6 h to 8 h with rotation.

Low salt buffer was used to wash the samples 3 times. (Low Salt Buffer contains 0.05 M HEPES-KOH, 0.15 M NaCl, 1 mM EDTA, 1% Triton X-100, 0.1% Sodium Deoxycholate, and 0.1% SDS.) High Salt Buffer was used to wash the samples twice. (The High Salt Buffer contains 0.05 M HEPES-KOH, 0.35 M NaCl, 1 mM EDTA, 1% Triton X-100, 0.1% Sodium Deoxycholate, and 0.1% SDS.) The samples were then washed once with LiCl Buffer. (The LiCl Buffer contains 0.01 M Tris-HCl, 0.25 M LiCl, 1 mM EDTA, 2.5% NP-40, and 0.5% Sodium Deoxycholate.) The samples were washed once with TE Buffer before resuspending the magnetic beads in 1ml of TE Buffer. After discarding the TE Buffer, the magnetic beads were resuspended in ChIP Elution Buffer to elute the DNA. (The ChIP Elution Buffer contains 0.05 M Tris-HCl, 0.01 M EDTA, and 1% SDS.) The eluted DNA was decrosslinked at 55 °C for 6 to 8 h. After decrosslinking, a phenol/chloroform/isoamyl alcohol (25:24:1) solution was added to the sample and mixed well. The sample was then transferred to centrifuged MaXtract High-Density tubes and centrifuged at 13,000 r/min for 5 min at room temperature. The supernatant was transferred to a new centrifuge tube, and sodium acetate, GlycoBlue, and prechilled isopropanol were added to purify the DNA. The purified DNA was prepared for sequencing using the NEB Next Ultra II DNA Library Prep Kit for Illumina kit. The PCR products were sorted by AMPure XP magnetic beads according to the length of the library fragments. The concentration of the mixed magnetic beads was 0.55× and 0.2×, and PE150 sequencing was performed with the Illumina HiSeq X Ten.

We utilized the FastQC software tool to analyze the characteristics of the library data. To remove linkers and low-quality sequences, we employed Trimmomatic software tools [43]. For alignment of the library fragments to the reference genome, we utilized the Burrow-Wheeler Aligner’s mem algorithm module [47]. Fragments with low alignment quality were filtered out from the library using the view module of SAMtools [48]. A script was utilized to call the view module of Sambamba software and retain uniquely aligned library fragments [45]. Duplicate sequences were removed from the library using Picard’s MarkDuplicates module. An index of the alignment result files was created using the index module of SAMtools [48]. For the calculation of small-volume alignment results files, we used the bamCoverage module of deepTools [49] and visualized the data with the Integrative Genomics Viewer [50]. The multiBamSummary module of deepTools was used to count differences between datasets [49]. To detect signals, we utilized MACS2 callpeak [51]. RNAPII recruitment has a broad peak shape, while H3K4me3 and H3K9ac have narrow peaks. To annotate peaks, we used the annotatePeaks.pl script from Homer [52]. To create annotation files for RNAPII, H3K4me3, and H3K9ac genome-wide peaks, we employed the intersect module of deepTools and a script [49]. The peak signal intensity was counted based on the peak annotation file using Htseq’s htseq-count module [53].

### ATAC-seq and data analysis

We infiltrated the sample with 1× PBS solution (including 1× PI) in a small petri dish and homogenized it by repeatedly cutting it with a blade. The sample was then filtered twice through a single-layer membrane, and 20ml of the filtrate was collected. We used the nonfixed angle accessory of a sizeable refrigerated centrifuge to centrifuge the sample at 1000 G for 5 min at 4 °C. After removing the supernatant, we washed the pellet twice with 1× PBS solution (containing 1× PI) and then resuspended it in 10 ml of 1× PBS solution (containing 1× PI). Next, we washed the pellet once more with Transposase Incubation Buffer, and after each wash, we centrifuged it at 600G for 3 min at 4 °C and discarded the supernatant. (Transposase Incubation Buffer: 0.02M HEPES Buffer (pH 7.5), 0.3M NaCl, 0.5 µM Spermidine, 0.1 M MgCl_2_). To continue, we took an appropriate amount of cell nuclei and resuspended them in 500 µL of Tagmentation Buffer. The resuspended cell nuclei were incubated at a low temperature for 10 min and then treated in a shaking dry bath device at 37 °C for 30 min at 300 r/min. (Tagmentation Buffer: 492.5 µL Transposase Incubation Buffer, 7.5 µL 20% Triton X-100 containing 0.1 M MgCl_2_). DNA was purified using the QIAGEN MinElute PCR Purification Kit. The purified DNA was then prepared using the NEB Next Ultra II DNA Library Prep Kit for Illumina kit, and the PCR products were sorted by AMPure XP magnetic beads. The concentrations of the mixed magnetic beads were 0.55× and 0.2×, and the resulting library was subjected to PE150 sequencing using Illumina HiSeq X Ten.

We utilized the FastQC software tool to assess the data characteristics of the library. Subsequently, we employed Trimmomatic software tools to eliminate linkers and low-quality sequences from the library [43]. Burrow-Wheeler Aligner’s mem algorithm module aligned the library fragments to the reference genome [47]. To remove poorly aligned fragments, we used the view module of SAMtools [48], while uniquely aligned library fragments were retained using a script and by calling the view module of Sambamba software [45]. Duplicate sequences were removed from the library using Picard’s markduplicates module. We employed the index module of SAMtools to generate an index of the alignment result files [48]. Furthermore, we utilized the bamCoverage module of deepTools to compute alignment result files of small volumes, and visualized them using the Integrative Genomics Viewer [50]. MACS2 callpeak was employed to detect signals [51], whereas high-confidence peaks for technical replicates were obtained using IDR [54].

### Correlation analysis of biological replicates

The similarity matrix between biological replicates was calculated using the multiBamSummary module of deepTools [49]. Subsequently, a heatmap or scatter plot was generated using the plotCorrelation module to visualize the correlation between the replicates.

### Time-series analysis of diurnal oscillation

The local database was initially trained using Biocycle, and subsequently, a deep neural network was employed to calculate a data matrix, resulting in the derivation of time-series features for each gene [34, 35, 55]. Transcriptome data were normalized to the expression levels of protein-coding genes using the TPMCalculator, which calculates the expression values in transcripts per million (TPM) [46]. Meanwhile, the peak data associated with epigenetic modifications were normalized by the deepTools software package using bins per million mapped reads (BPM) to reflect the signal intensity of each peak [49]. Finally, the merged data generated a time-series matrix.

### Data visualization

The unique alignment result files from the ATAC-seq, ChIP-seq, and RNA-seq datasets were used to generate bigWig format files by applying the BPM calculation standard through the bamCoverage module of deepTools [49]. These bigWig files were then utilized for data visualization with IGV software [50].

### Supplementary Information

**Additional file 1:** **Fig S1.** Meteorological conditions during the two-day sampling period. **Fig S2.** Repeatability of sequencing data from technical replicates at the same time. **Fig S3.** Epigenetic modification characterization and annotation of rhythmic genes in *B. napus*. **Fig S4** Epigenetic modifications promote gene transcription in oscillation regulation. **Fig S5.** Cluster analysis of diurnal oscillation epigenetic modifications. **Fig S6.** Diurnal oscillation of H3K4me3. **Fig S7.** Diurnal oscillation of H3K9ac. Fig S8. Diurnal oscillation of RNAPII. **Fig S9.** Diurnal oscillatory properties of gene transcription concerning chromatin accessibility, subgenomic dominance, and sequence features. **Fig S10.** Diurnal oscillation differences in transcription of biological clock homologous genes on different subgenomes. **Fig S11.** Effects of epigenetic modifications on diurnal oscillations in gene transcription. **Fig S12.** Variability in rhythmic expression of biological clock homologous genes is associated with differences in diurnal oscillations of histone modification H3K9ac. **Fig S13.** Variations in rhythmic expression of biological clock homologous genes are associated with differences in diurnal oscillations in RNAPII recruitment. **Fig S14.** Combinations of multiple epigenetic modifications define diurnal oscillations of *RVE1*.

**Additional file 2:** **Supplementary Dataset 1.** Oscillating H3K4me3 peaks.

**Additional file 3:** **Supplementary Dataset 2.** Oscillating H3K9ac peaks.

**Additional file 4**: **Supplementary Dataset 3.** Oscillating RNAPII peaks.

**Additional file 5:** **Supplementary Dataset 4. **Oscillating biological clock genes.

**Additional file 6**: **Supplementary Dataset 5. **The oscillating characteristic of protein-coding gene transcription and their diurnal rhythm of epigenetic modifications.

## Data Availability

All data generated or analyzed during this study are included in this published article, its supplementary information files and publicly available repositories. The sequencing data generated in this study have been deposited in the National Genomics Data Center (NGDC) China National Center for Bioinformation (CNCB) Genome Sequence Archive database (GSA) under the accession number PRJCA010169 [56]. The diurnal oscillation gene expression dataset of *Brassica rapa* is derived from the published NCBI Gene Expression Omnibus with the accession number GSE90841 [57]. The supplementary figures and datasets of this article, serving as crucial outcomes in the data analysis process, have also been published on Figshare [58].

## Abbreviations

*B. napus*: *Brassica napus*
*B. rapa*: *Brassica rapa*
*B. oleracea*: *Brassica oleracea*
ChIA-PET: Chromatin Interaction Analysis with Paired-End Tag
Hi-C: High-throughput chromosome conformation capture
SCN: Suprachiasmatic nucleus
GO: Gene ontology
KEGG: Kyoto Encyclopedia of Genes and Genomes
TSS: Transcriptional start site
TPM: Transcripts per million
H3K4me3: Tri-methylation of histone H3 at lysine 4
H3K9ac: Acetylation of histone H3 at lysine 9
RNAPII: RNA polymerase II
ChIP: Chromatin immunoprecipitation
ATAC: Assay for transposase-accessible chromatin
