# Supplementary material for: Diurnal oscillations of epigenetic modifications are associated with variation in rhythmic expression of homoeologous genes in Brassica napus
Source: BMC Biol. 2023 Oct 31;21:241. doi: 10.1186/s12915-023-01735-7 (PMC10617162; doi:10.1186/s12915-023-01735-7)
Supplement: Supplementary file 1 — Additional file 1: Fig S1. Meteorological conditions during the two-day sampling period. Fig S2. Repeatability of sequencing data from technical replicates at the same time. Fig S3. Epigenetic modification characterization and annotation of rhythmic genes in B. napus. Fig S4 Epigenetic modifications promote gene transcription in oscillation regulation. Fig S5. Cluster analysis of diurnal oscillation epigenetic modifications. Fig S6. Diurnal oscillation of H3K4me3. Fig S7. Diurnal oscillation of H3K9ac. Fig S8. Diurnal oscillation of RNAPII. Fig S9. Diurnal oscillatory properties of gene transcription concerning chromatin accessibility, subgenomic dominance, and sequence features. Fig S10. Diurnal oscillation differences in transcription of biological clock homologous genes on different subgenomes. Fig S11. Effects of epigenetic modifications on diurnal oscillations in gene transcription. Fig S12. Variability in rhythmic expression of biological clock homologous genes is associated with differences in diurnal oscillations of histone modification H3K9ac. Fig S13. Variations in rhythmic expression of biological clock homologous genes are associated with differences in diurnal oscillations in RNAPII recruitment. Fig S14. Combinations of multiple epigenetic modifications define diurnal oscillations of RVE1. [file 12915_2023_1735_MOESM1_ESM.docx]

**Supplemental Information for**

**Diurnal oscillations of epigenetic modifications are associated with variation in rhythmic expression of homoeologous genes in *Brassica napus***

Zhifei Xue^1^, Baibai Gao^1^, Guoting Chen^1^, Jie Liu^2^, Weizhi Ouyang^1^, Mohamed Frahat Foda^1,3^, Qing Zhang^1^, Xiwen Zhang^1^, Wei Zhang^1^, Mingyue Guo^4^, Xingwang Li^1,*^ and Bin Yi^1,5,*^

^1^National Key Laboratory of Crop Genetic Improvement, Hubei Hongshan Laboratory, Huazhong Agricultural University, Wuhan, Hubei 430070, China

^2^Lushan Botanical Garden Jiangxi Province and Chinese Academy of Sciences, Jiujiang, Jiangxi 332900, China

^3^Department of Biochemistry, Faculty of Agriculture, Benha University, Moshtohor, Toukh13736, Egypt

^4^College of Informatics, Huazhong Agricultural University, Wuhan, Hubei 430070, China

^5^National Engineering Research Center of Rapeseed, Huazhong Agricultural University, Wuhan, Hubei 430070, China

***Correspondence:** Xingwang Li (xingwangli@mail.hzau.edu.cn) & Bin Yi (yibin@mail.hzau.edu.cn)

# Figure S1.


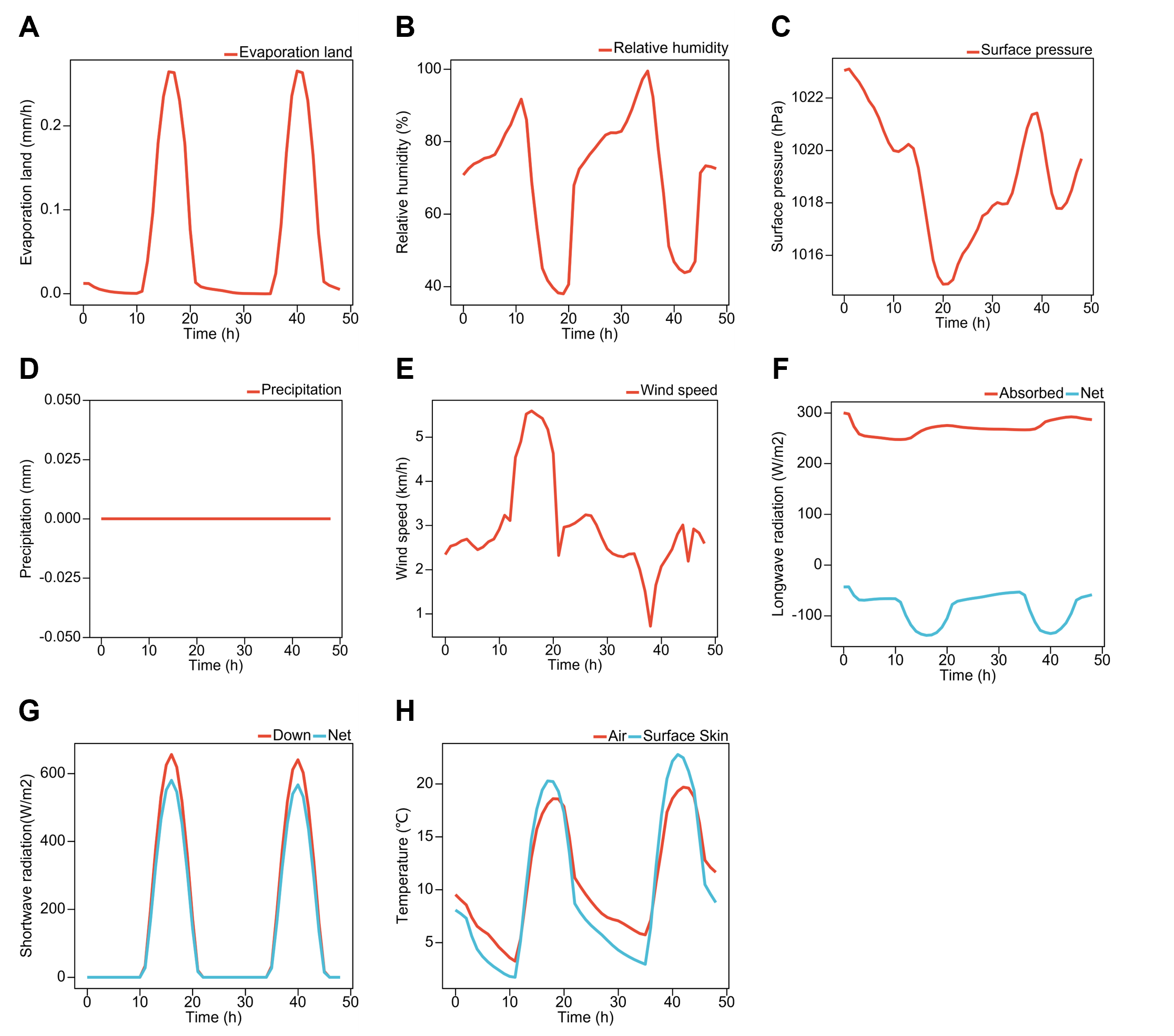


**Fig S1. Meteorological conditions during the two-day sampling period.**

(A) Surface evaporation (mm/h). (B) Relative humidity (%). (C) Surface atmospheric pressure (hPa). (D) Precipitation (mm). (E) Wind speed (km/h). (F) Longwave radiation (W/m2). (G) Shortwave radiation (W/m2). (H) Atmospheric and surface temperatures (°C). Meteorological data were measured at hourly intervals.

# Figure S2.

**
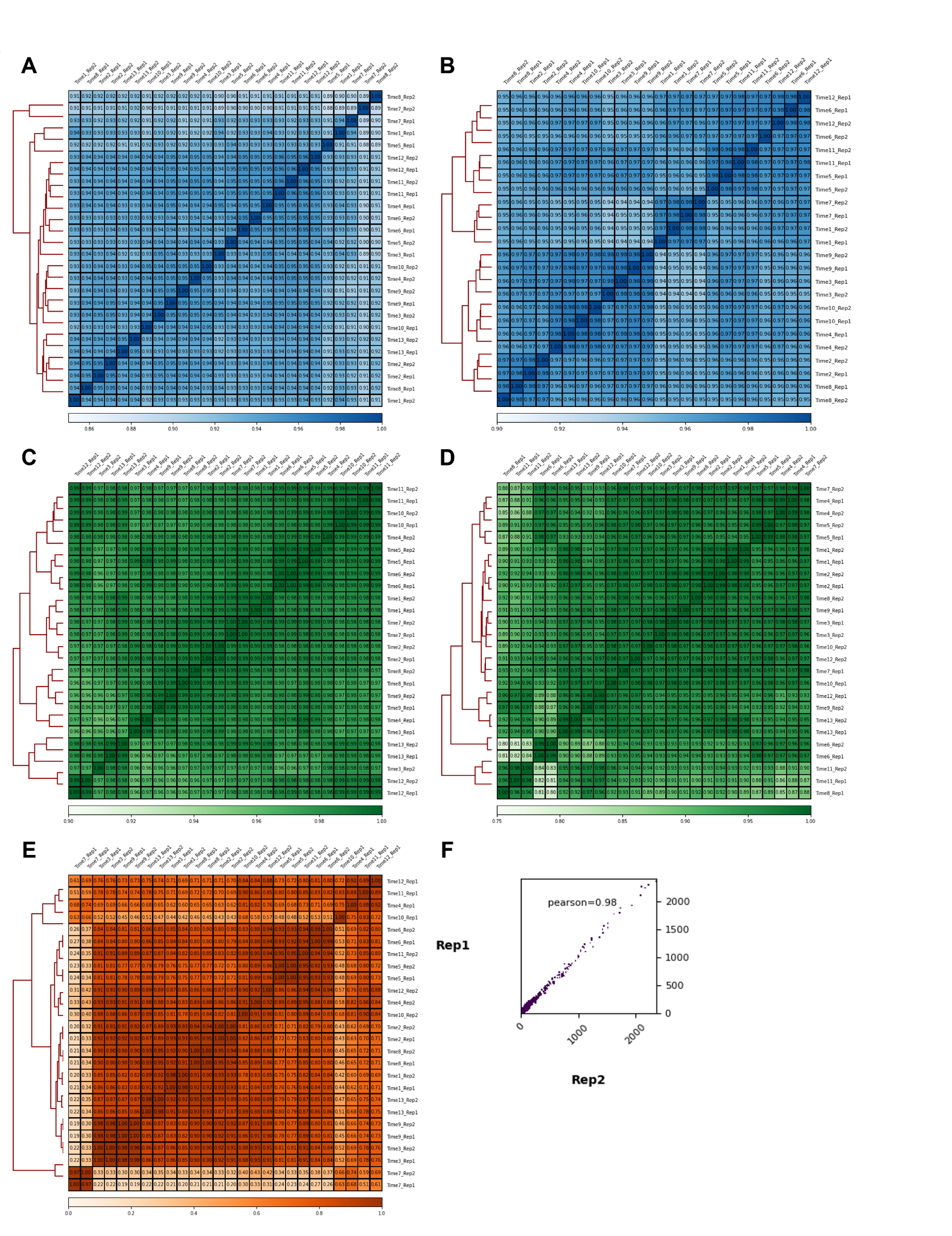
**

**Fig S2. Repeatability of sequencing data from technical replicates at the same time.**

(A) Repeatability of *B. napus* transcriptome at 13 time points over two days. (B) Repeatability of *B. rapa* transcriptome data at 12 time points over two days. (C-E) Repeatability of ChIP-seq data of histone modifications H3K4me3, H3K9ac, and RNAPII recruitment at 13 time points in *B. napus* over 2 days. (F) Repeatability of ATAC-seq at the seedling stage of *B. napus*.

# Figure S3.

**
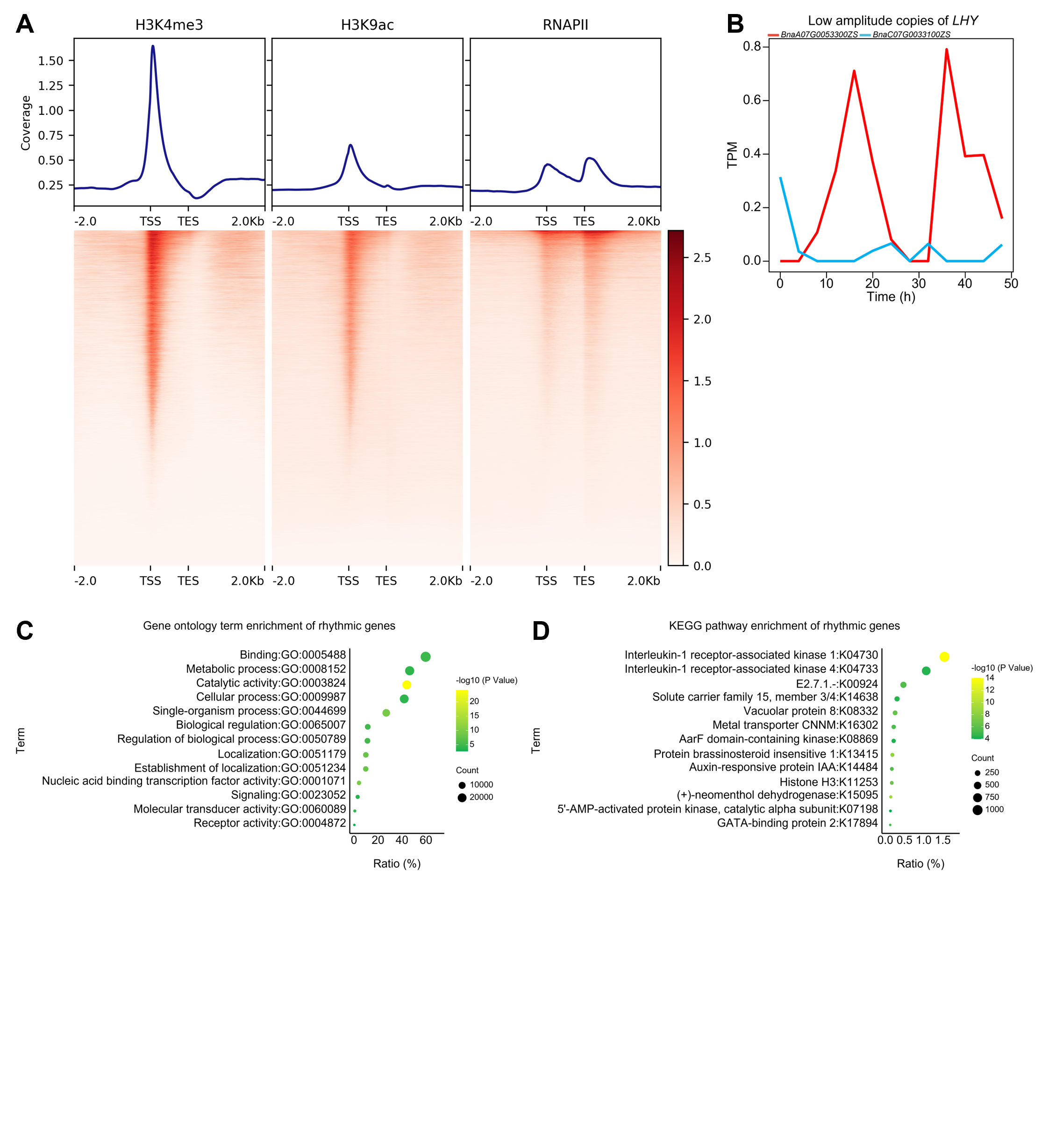
**

**Fig S3. Epigenetic modification characterization and annotation of rhythmic genes in *B. napus*.**

(A) Distribution of histone modifications H3K4me3, H3K9ac, and RNAPII recruitment on genes in *B. napus*. (B) Diurnal fluctuations in low-amplitude homologous gene expression of *LHY*. (C) Gene Ontology (GO) annotation of rhythmic genes. (D) KEGG enrichment analysis of rhythmic genes.

# Figure S4.

**
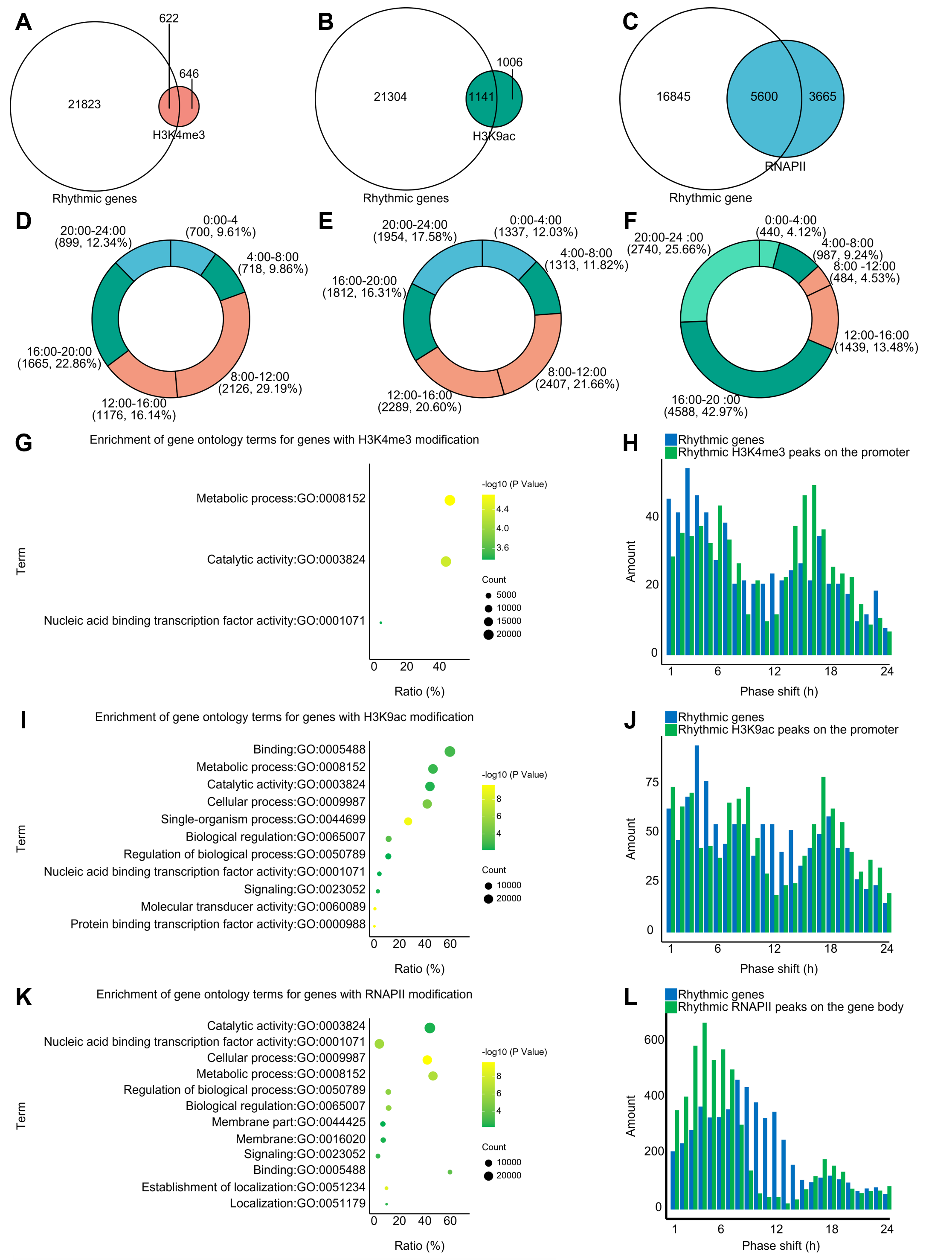
**

**Fig S4. Epigenetic modifications promote gene transcription in oscillation regulation.**

(A) Overlap between genes exhibiting diurnal oscillation in histone modification H3K4me3 and rhythmic genes. (B) Overlap between genes exhibiting diurnal oscillation in histone modification H3K9ac and rhythmic genes. (C) Overlap between genes exhibiting diurnal oscillation in RNAPII recruitment and rhythmic genes. (D) Peak phase analysis of diurnal oscillatory H3K4me3 peaks. (E) Peak phase analysis of diurnal oscillatory H3K9ac peaks. (F) Peak phase analysis of diurnal oscillatory RNAPII peaks. (G) GO annotation of genes exhibiting diurnal oscillatory histone modification H3K4me3. (H) Phase analysis of gene epigenetic modification and transcription of H3K4me3 in the presence of diurnal oscillatory histone modifications at the promoter. (I) GO annotation of genes exhibiting diurnal oscillatory histone modification H3K9ac. (J) Phase analysis of gene epigenetic modification and transcription of histone modification H3K9ac in the presence of diurnal oscillatory promoters. (K) GO annotation of genes exhibiting diurnal oscillatory RNAPII recruitment. (L) Phase analysis of gene epigenetic modification and transcription in the presence of diurnal oscillatory RNAPII recruitment in the gene body.

Figure S5.

**
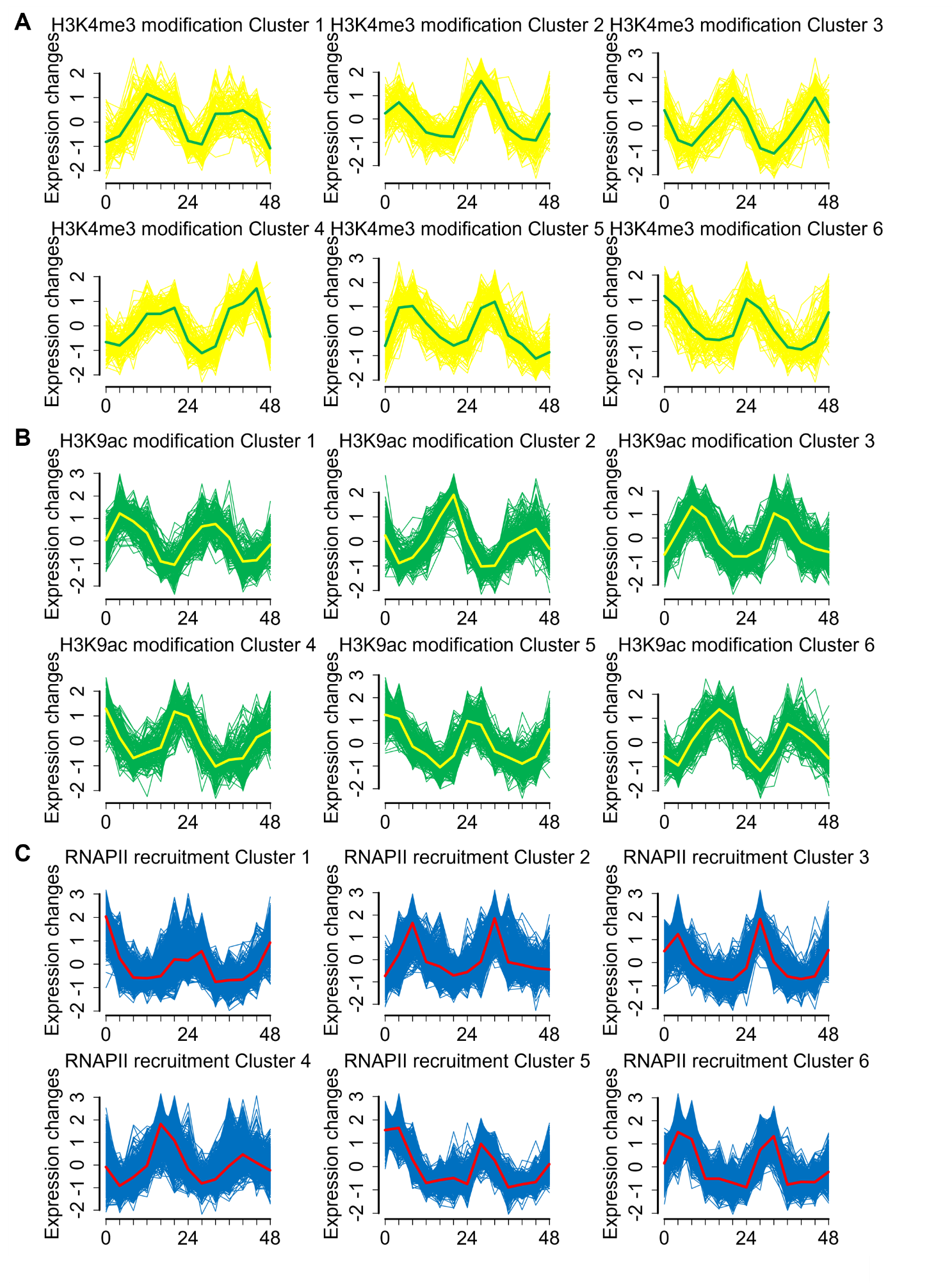
**

**Fig S5. Cluster analysis of diurnal oscillation epigenetic modifications.**

(A-C) Cluster analysis of diurnal oscillating histone modified of H3K4me3, H3K9ac, and RNAPII recruitment.

Figure S6.

**
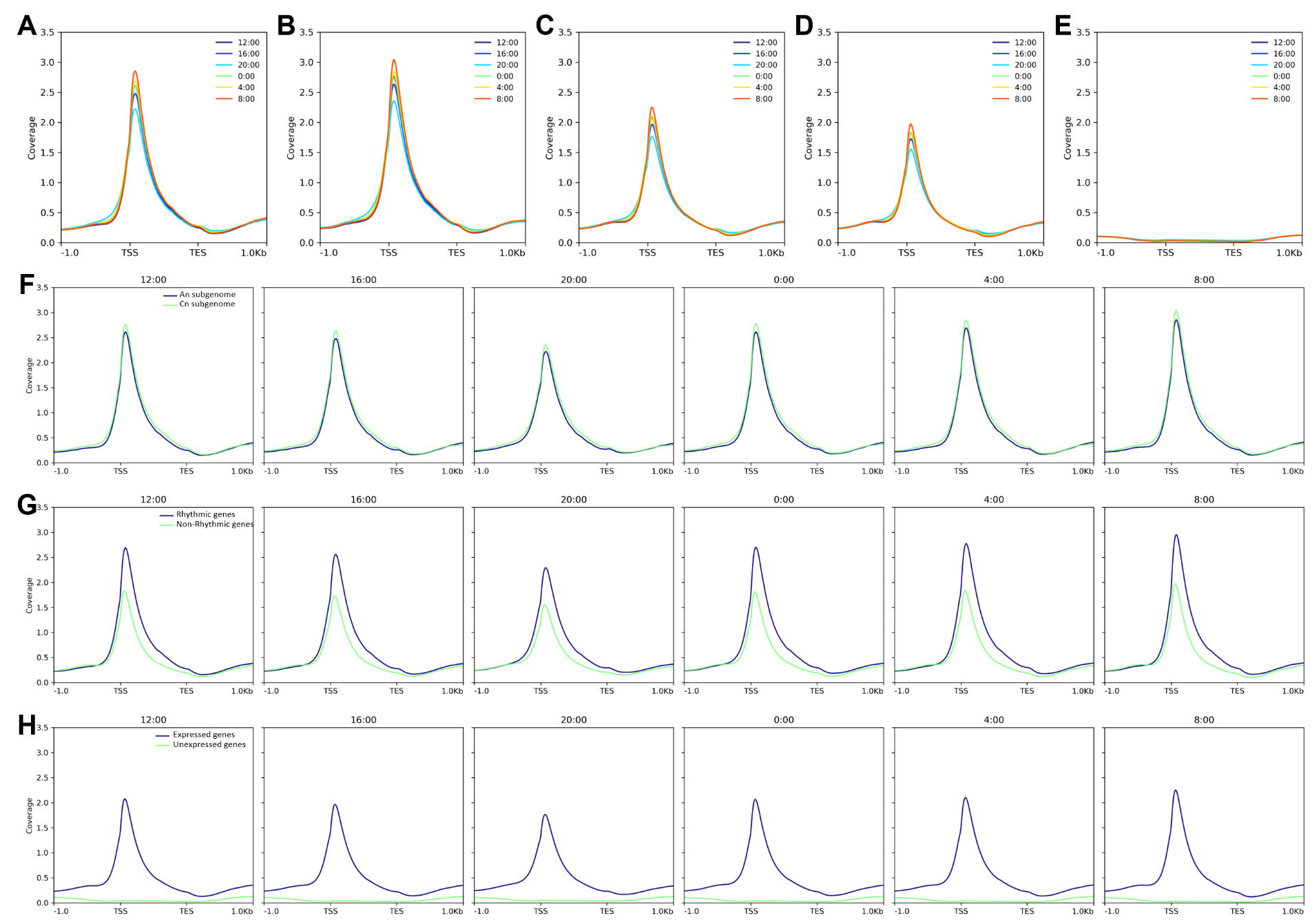
**

**Fig S6. Diurnal oscillation of H3K4me3.**

(A-E) Binding profiles of H3K4me3 at the gene body of rhythmic genes on the An subgenome, rhythmic genes on the Cn subgenome, expressed genes, non-rhythmic genes, and unexpressed genes. (F) Comparison of binding profiles of H3K4me3 in rhythmic genes between the An and Cn subgenome. (G) Comparison of binding profiles of H3K4me3 between rhythmic and non-rhythmic genes. (H) Comparison of binding profiles of H3K4me3 between expressed and unexpressed genes.

Figure S7.

**
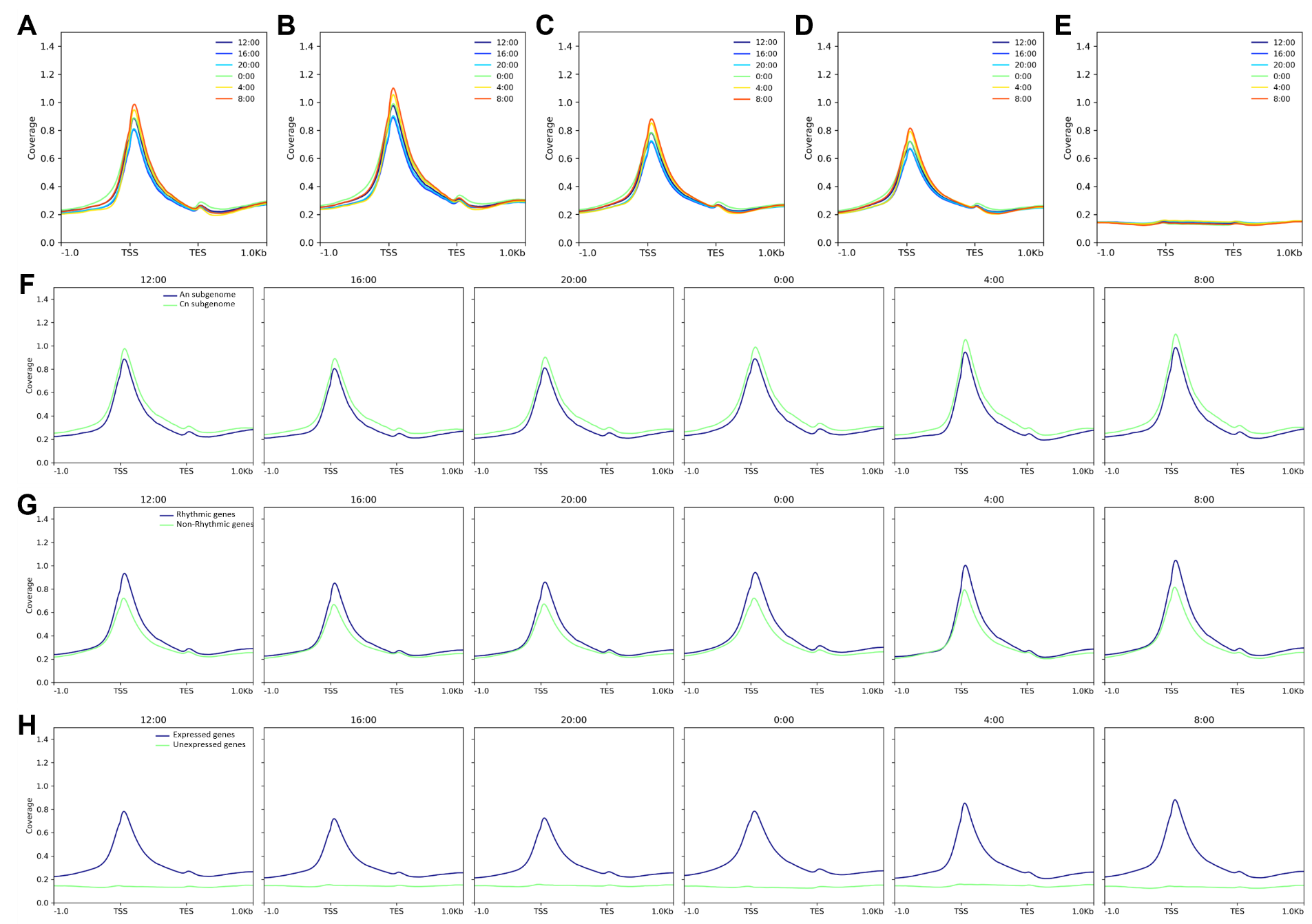
**

**Fig S7. Diurnal oscillation of H3K9ac.**

(A-E) Binding profiles of H3K9ac at the gene body of rhythmic genes on the An subgenome, rhythmic genes on the Cn subgenome, expressed genes, non-rhythmic genes, and unexpressed genes. (F) Comparison of binding profiles of H3K9ac in rhythmic genes between the An and Cn subgenome. (G) Comparison of binding profiles of H3K9ac between rhythmic and non-rhythmic genes. (H) Comparison of binding profiles of H3K9ac between expressed and unexpressed genes.

Figure S8.

**
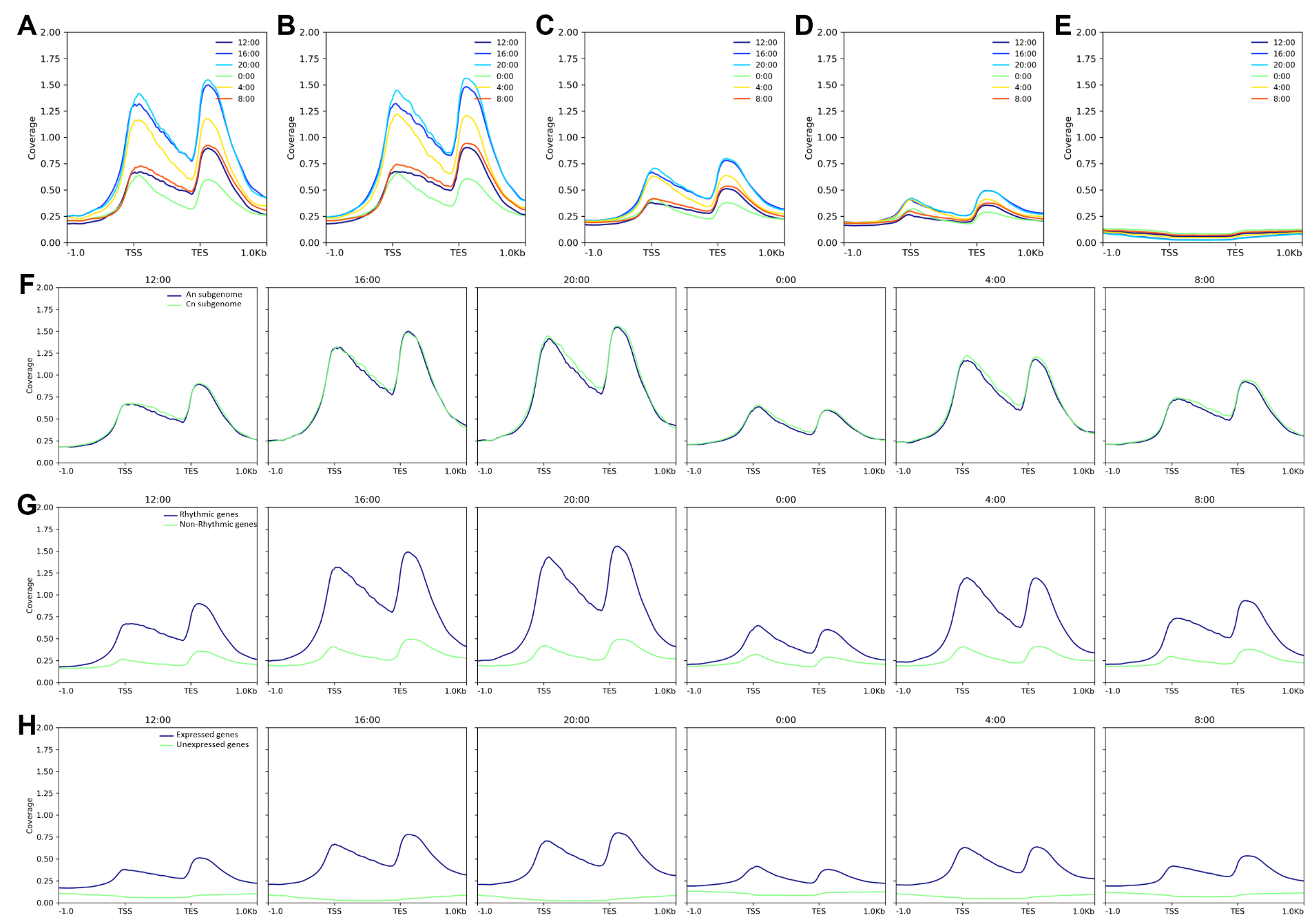
**

**Fig S8. Diurnal oscillation of RNAPII.**

(A-E) Binding profiles of RNAPII at the gene body of rhythmic genes on the An subgenome, rhythmic genes on the Cn subgenome, expressed genes, non-rhythmic genes, and unexpressed genes. (F) Comparison of binding profiles of RNAPII in rhythmic genes between the An and Cn subgenome. (G) Comparison of binding profiles of RNAPII between rhythmic and non-rhythmic genes. (H) Comparison of binding profiles of RNAPII between expressed and unexpressed genes.

Figure S9.

**
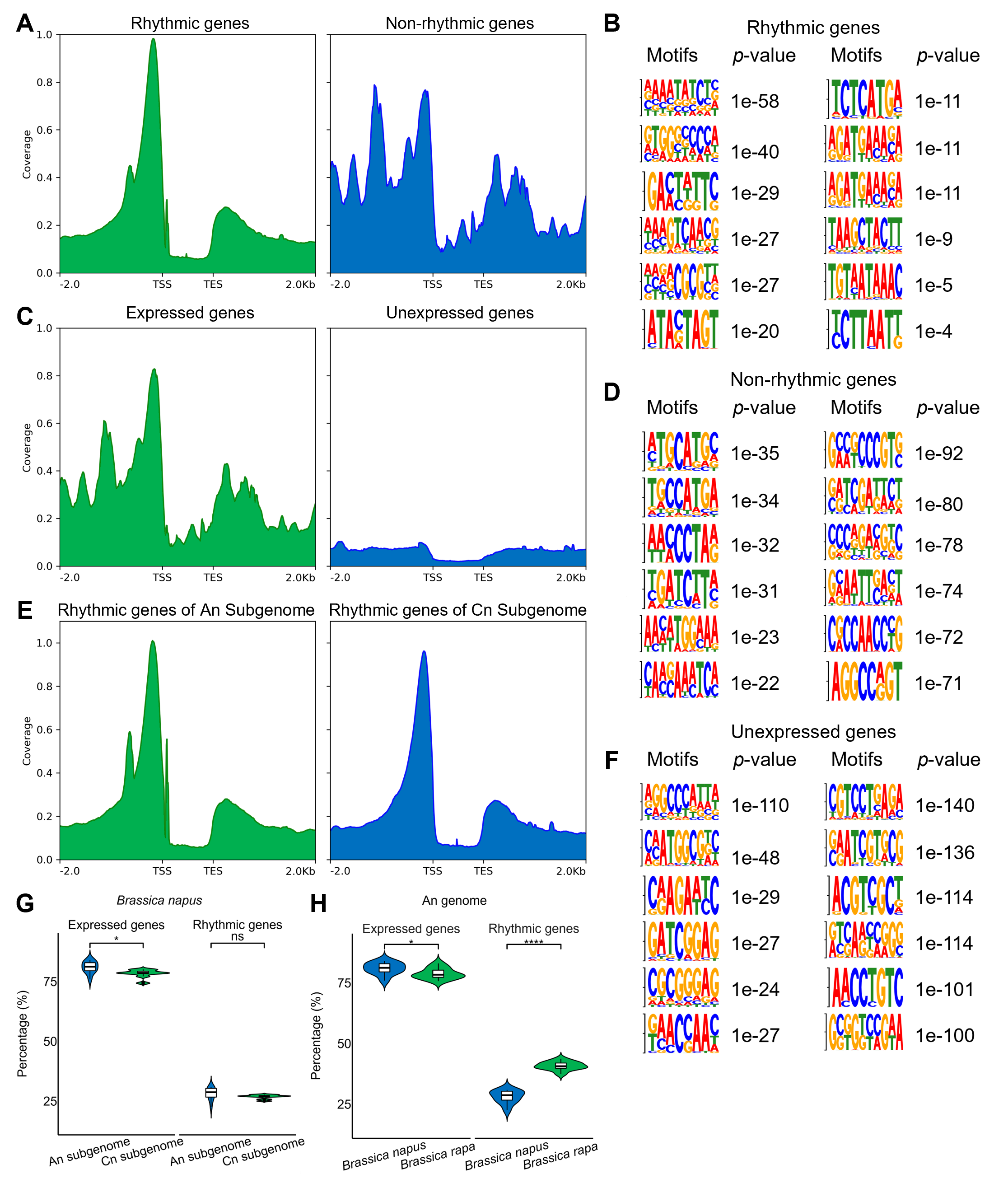
**

**Fig S9. Diurnal oscillatory properties of gene transcription concerning chromatin accessibility, subgenomic dominance, and sequence features.**

(A) Comparison of chromatin accessibility between rhythmic and non-rhythmic genes (B) Motif analysis of rhythmic genes. (C) Comparison of chromatin accessibility between expressed and unexpressed genes. (D) Motif analysis of non-rhythmic genes. (E) Comparison of chromatin accessibility of rhythmic genes between the An and Cn subgenomes. (F) Motif analysis of unexpressed genes. (G) Significance analysis of differences in the distribution of rhythmic and non-rhythmic genes between the An and Cn subgenomes. (H) Significant differences in the distribution of rhythmic and non-rhythmic genes on the A genomes between *B. napus* and *B. rapa*.

Figure S10.

**
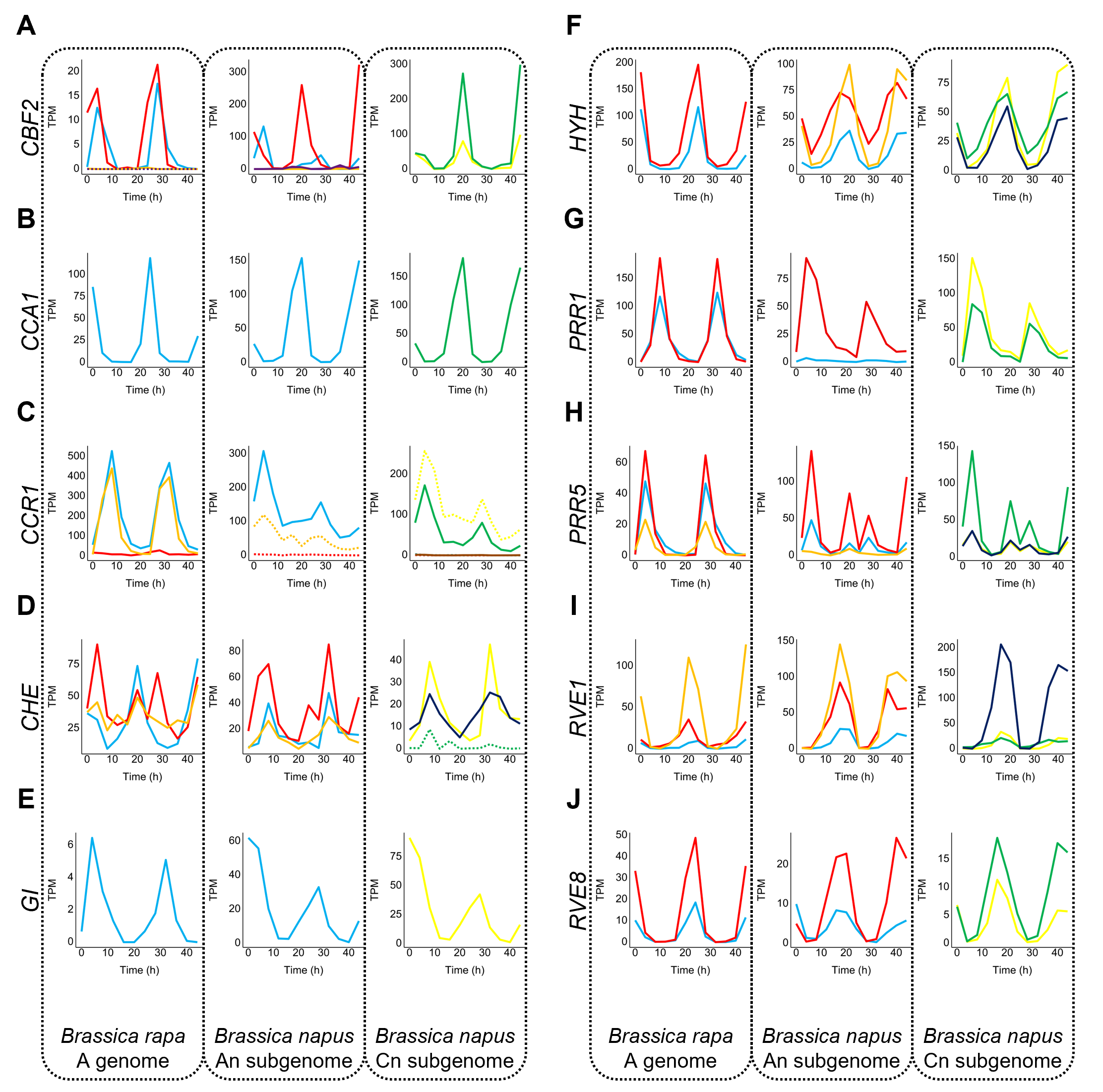
**

**Fig S10. Diurnal oscillation differences in transcription of biological clock homologous genes on different subgenomes.**

(A-J) Transcriptional profiles of homologous genes of *CBF2*, *CCA1*, *CCR1*, *CHE*, *GI*, *HYH*, *PRR1*, *PRR5*, *RVE1*, and *RVE8* in the A genome of *B. rapa*, and the An and Cn subgenomes of *B. napus*, respectively. Displaying only rhythmic genes within homologous copies, with different colors used to distinguish multiple homologous copies.

Figure S11.

**
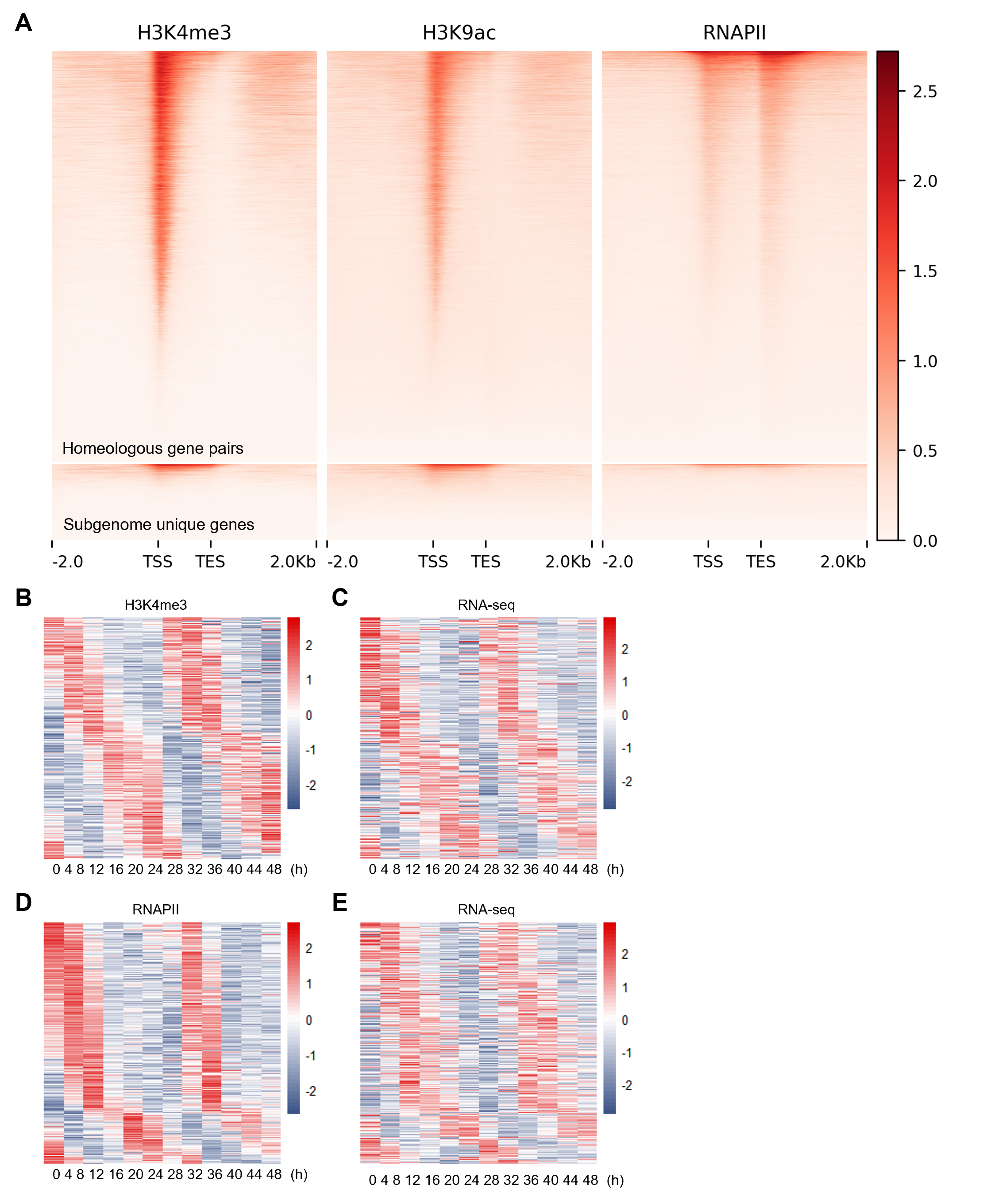
**

**Fig S11. Effects of epigenetic modifications on diurnal oscillations in gene transcription.**

(A) Differences in epigenetic modifications between homologous gene pairs and subgenome-specific genes. (B) Diurnal oscillation of histone modification H3K4me3. (C) Rhythmic expression of genes with diurnal oscillating H3K4me3 peaks. (D) Diurnal oscillations of RNAPII recruitment. (E) Rhythmic expression of genes with diurnal oscillating RNAPII peaks.

Figure S12.

**
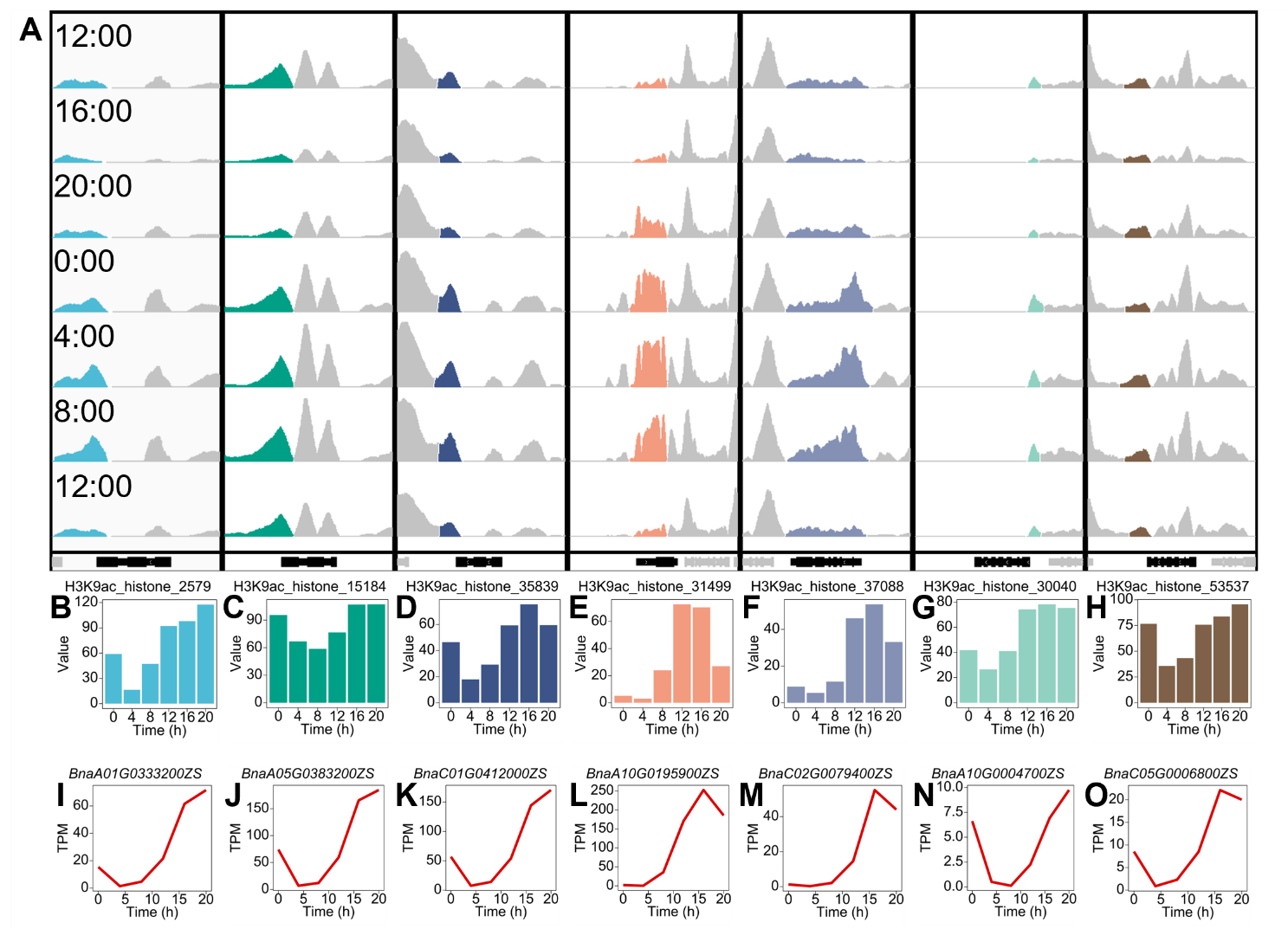
**

**Fig S12. Variability in rhythmic expression of biological clock homologous genes is associated with differences in diurnal oscillations of histone modification H3K9ac.**

(A) Features of diurnal oscillation of histone modification H3K9ac on *HYH* (*BnaA01G0333200ZS*, *BnaA05G0383200ZS*, and *BnaC01G0412000ZS*), *RVE1* (*BnaA10G0195900ZS* and *BnaC02G0079400ZS*), and *RVE3* (*BnaA10G0004700ZS* and *BnaC05G0006800ZS*). (B-H) Diurnal oscillation of histone modification H3K9ac on *HYH* (*BnaA01G0333200ZS*, *BnaA05G0383200ZS*, and *BnaC01G0412000ZS*), *RVE1* (*BnaA10G0195900ZS* and *BnaC02G0079400ZS*), and *RVE3* (*BnaA10G0004700ZS* and *BnaC05G0006800ZS*). (I-O) Diurnal fluctuations in homologous gene expression of *HYH* (*BnaA01G0333200ZS*, *BnaA05G0383200ZS*, and *BnaC01G0412000ZS*), *RVE1* (*BnaA10G0195900ZS* and *BnaC02G0079400ZS*), and *RVE3* (*BnaA10G0004700ZS* and *BnaC05G0006800ZS*).

Figure S13.

**
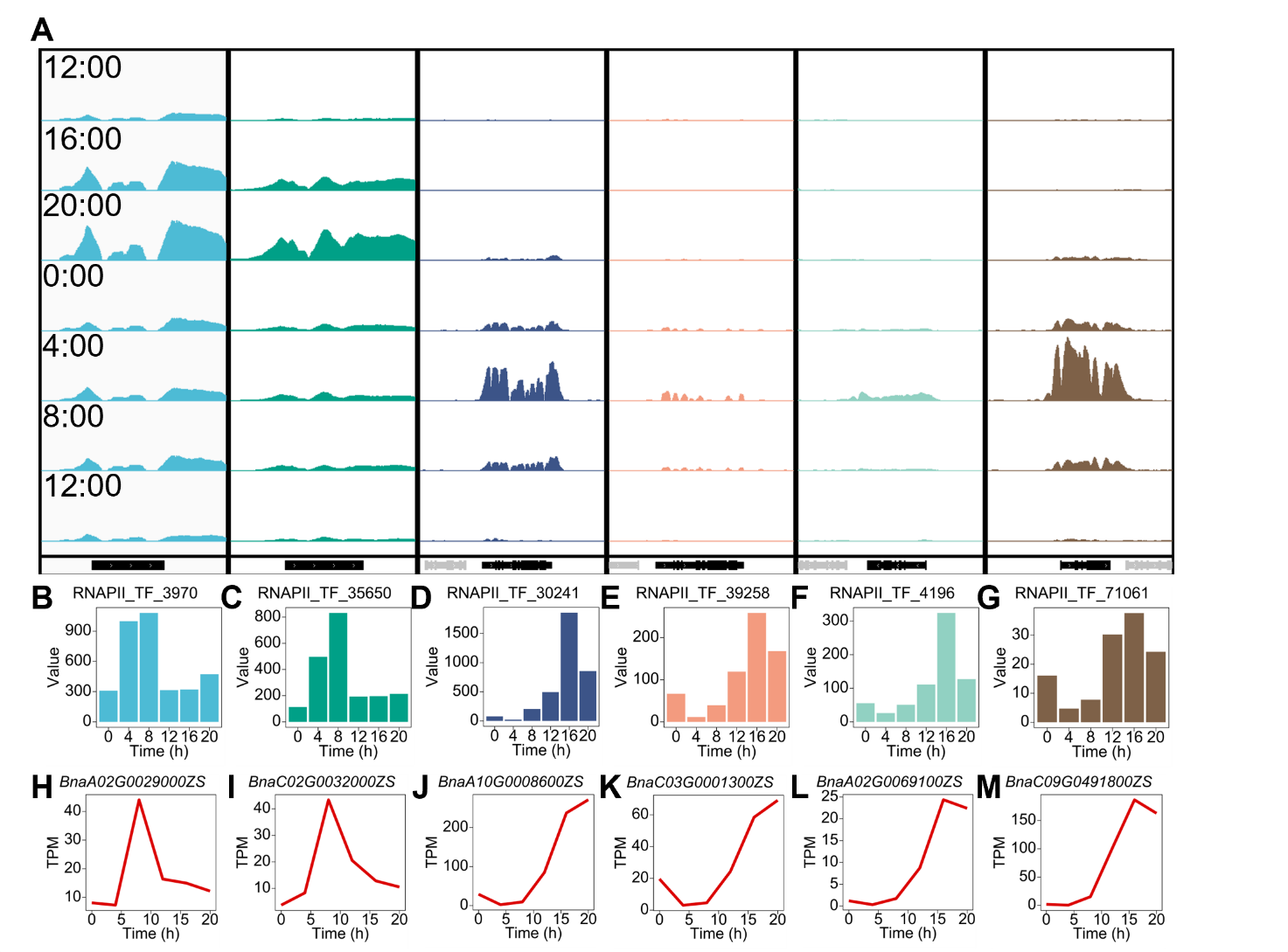
**

**Fig S13. Variations in rhythmic expression of biological clock homologous genes are associated with differences in diurnal oscillations in RNAPII recruitment.**

(A) Features of diurnal oscillation of histone modification RNAPII recruitment on *CHE* (*BnaA02G0029000ZS* and *BnaC02G0032000ZS*), *LHY* (*BnaA10G0008600ZS* and *BnaC03G0001300ZS*), and *RVE1* (*BnaA02G0069100ZS* and *BnaC09G0491800ZS*) homologous genes. (B-G) Diurnal oscillation of RNAPII recruitment on *CHE* (*BnaA02G0029000ZS* and *BnaC02G0032000ZS*), *LHY* (*BnaA10G0008600ZS* and *BnaC03G0001300ZS*), and *RVE1* (*BnaA02G0069100ZS* and *BnaC09G0491800ZS*) homologous genes. (H-M) Diurnal fluctuations in homologous gene expression of *CHE* (*BnaA02G0029000ZS* and *BnaC02G0032000ZS*), *LHY* (*BnaA10G0008600ZS* and *BnaC03G0001300ZS*), and *RVE1* (*BnaA02G0069100ZS* and *BnaC09G0491800ZS*) homologous genes.

Figure S14.

**
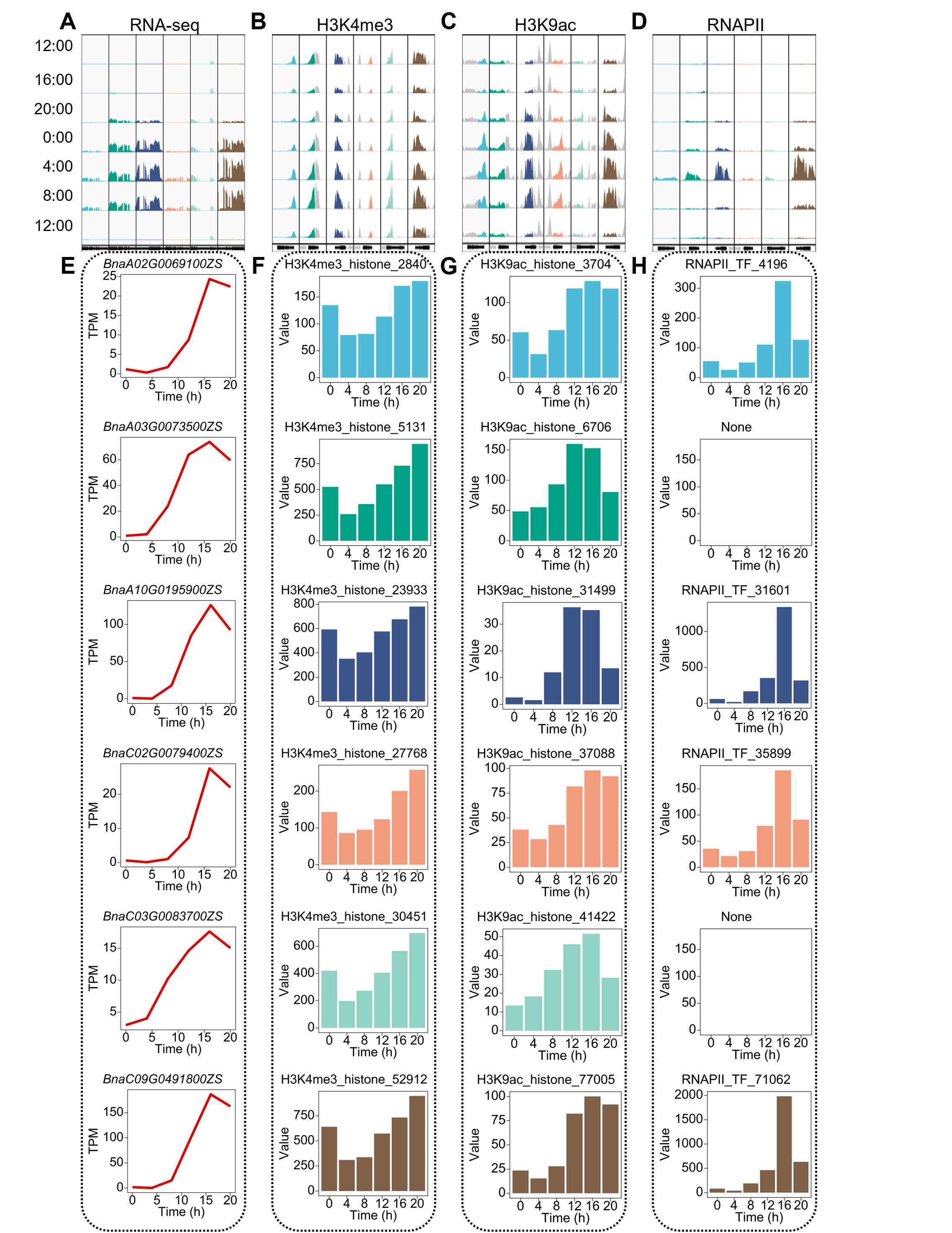
**

**Fig S14. Combinations of multiple epigenetic modifications define diurnal oscillations of *RVE1*.**

(A) Features of diurnal gene expression oscillation in 6 homologous genes of *RVE1*. (B) Features of diurnal histone modification H3K4me3 oscillation in 6 homologous genes of *RVE1*. (C) Features of diurnal histone modification H3K9ac oscillation in 6 homologous genes of *RVE1*. (D) Features of diurnal RNAPII recruitment oscillation in 6 homologous genes of *RVE1*. (E) Diurnal oscillation of expression in 6 homologous genes of *RVE1*. (F) Diurnal oscillation of histone modification H3K4me3 in 6 homologous genes of *RVE1*. (G) Diurnal oscillation of histone modification H3K9ac in 6 homologous genes of *RVE1*. (H) Diurnal oscillations of RNAPII recruitment in 6 homologous genes of *RVE1*.
